# Nanoliposomes as nonviral vectors in cancer gene therapy

**DOI:** 10.1002/mco2.583

**Published:** 2024-06-25

**Authors:** Safiye Nur Yildiz, Maliheh Entezari, Mahshid Deldar Abad Paskeh, Sepideh Mirzaei, Alireza Kalbasi, Amirhossein Zabolian, Farid Hashemi, Kiavash Hushmandi, Mehrdad Hashemi, Mehdi Raei, Mohammad Ali Sheikh Beig Goharrizi, Amir Reza Aref, Ali Zarrabi, Jun Ren, Gorka Orive, Navid Rabiee, Yavuz Nuri Ertas

**Affiliations:** ^1^ Department of Biomedical Engineering Erciyes University Kayseri Turkey; ^2^ Department of Genetics Faculty of Advanced Science and Technology Tehran Medical Sciences Islamic Azad University Tehran Iran; ^3^ Department of Medical Convergence Sciences Farhikhtegan Hospital Tehran Medical Sciences Islamic Azad University Tehran Iran; ^4^ Department of Biology Faculty of Science Islamic Azad University Science and Research Branch Tehran Iran; ^5^ Department of Pharmacy Brigham and Women's Hospital Harvard Medical School Boston Massachusetts USA; ^6^ Department of Orthopedics Shahid Beheshti University of Medical Sciences Tehran Iran; ^7^ Department of Comparative Biosciences Faculty of Veterinary Medicine University of Tehran Tehran Iran; ^8^ Department of Clinical Sciences Institute Nephrology and Urology Research Center Baqiyatallah University of Medical Sciences Tehran Iran; ^9^ Department of Epidemiology and Biostatistics School of Health Baqiyatallah University of Medical Sciences Tehran Iran; ^10^ Department of Biotechnology College of Science, University of Tehran Tehran Iran; ^11^ Belfer Center for Applied Cancer Science Dana‐Farber Cancer Institute Harvard Medical School Boston Massachusetts USA; ^12^ Department of Translational Sciences Xsphera Biosciences Inc. Boston Massachusetts USA; ^13^ Department of Biomedical Engineering Faculty of Engineering and Natural Sciences Istinye University Istanbul Turkey; ^14^ Shanghai Institute of Cardiovascular Diseases Department of Cardiology Zhongshan Hospital Fudan University Shanghai China; ^15^ NanoBioCel Research Group School of Pharmacy University of the Basque Country (UPV/EHU) Vitoria‐Gasteiz Spain; ^16^ University Institute for Regenerative Medicine and Oral Implantology ‐ UIRMI (UPV/EHU‐Fundación Eduardo Anitua) Vitoria‐Gasteiz Spain; ^17^ Bioaraba, NanoBioCel Research Group Vitoria‐Gasteiz Spain; ^18^ The Academia Singapore Eye Research Institute Singapore Singapore; ^19^ Centre for Molecular Medicine and Innovative Therapeutics Murdoch University Perth Western Australia Australia; ^20^ ERNAM—Nanotechnology Research and Application Center Erciyes University Kayseri Turkey; ^21^ UNAM−National Nanotechnology Research Center Bilkent University Ankara Turkey

**Keywords:** CRISPR/Cas9, liposome, nonviral vector, shRNA, siRNA

## Abstract

Nonviral vectors, such as liposomes, offer potential for targeted gene delivery in cancer therapy. Liposomes, composed of phospholipid vesicles, have demonstrated efficacy as nanocarriers for genetic tools, addressing the limitations of off‐targeting and degradation commonly associated with traditional gene therapy approaches. Due to their biocompatibility, stability, and tunable physicochemical properties, they offer potential in overcoming the challenges associated with gene therapy, such as low transfection efficiency and poor stability in biological fluids. Despite these advancements, there remains a gap in understanding the optimal utilization of nanoliposomes for enhanced gene delivery in cancer treatment. This review delves into the present state of nanoliposomes as carriers for genetic tools in cancer therapy, sheds light on their potential to safeguard genetic payloads and facilitate cell internalization alongside the evolution of smart nanocarriers for targeted delivery. The challenges linked to their biocompatibility and the factors that restrict their effectiveness in gene delivery are also discussed along with exploring the potential of nanoliposomes in cancer gene therapy strategies by analyzing recent advancements and offering future directions.

## Graphical abstract

Cancer gene therapy is a promising approach; however, more investigations are needed to improve its efficiency, liposomes are nanocarriers for drug and gene delivery, and display enhanced intracellular accumulation, targeted delivery and high biocompatibility. Their surface modification of nanoparticles by ligands enhances selectivity of gene delivery to cancer cells.

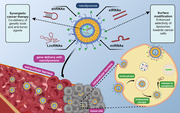

## 1 INTRODUCTION

Cancer treatment is a very challenging process that requires the application of pharmacological and genetic interventions to combat the disease. Such treatments are designed to target the underlying mechanisms of cancer cells, with the goal of inhibiting their growth and proliferation. These interventions can be delivered through a variety of approaches, including chemotherapy, radiation therapy, immunotherapy, and gene therapy. Gene therapy stands out among these methods and aims to prevent or treat various diseases by correcting or replacing mutated genes that cause specific disorders. Gene therapy has been a topic of interest since the 1960s and has made significant progress since then.^1^ Gene therapy has enormous potential shortly and is poised to become a significant field in healthcare. Recent developments in molecular biology, particularly in viral vectors and genome‐editing techniques, have significantly enhanced the accuracy and effectiveness of gene therapy interventions.^2^, ^3^, ^4^, ^5^ This has resulted in previously incurable genetic disorders being treated with potential hope for patients and their families. However, developing safe and effective gene therapies has been challenging. Delivering gene therapy to target cells is a significant hurdle researchers must overcome. It requires a delivery system that can transport the therapeutic gene to the target cells without causing any adverse effects. Additionally, the potential for off‐target effects and the risk of immunogenicity must be considered carefully. Despite these challenges, the benefits of gene therapy are vast, and the field is rapidly advancing.^6^

In the context of cancer treatment, the application of genetic intervention typically involves the use of numerous agents. Small molecule agents such as cisplatin (CP), doxorubicin, and temozolomide suffer from drug resistance, making them unsuitable agents to suppress for some cancer types.^7^, ^8^ Therefore, efforts have been directed toward using genetic interventions to target genes responsible for cancer growth and malignancy.^9^, ^10^, ^11^ Various genetic tools, including siRNA, shRNA, and the clustered regularly interspaced short palindromic repeats (CRISPR)/Cas9 system, are utilized for targeting genes in cancer therapy. There are, however, limitations associated with the aforementioned tools, such as their off‐targeting effects, degradation by enzymes and the presence of physiological impediments represented by the blood–brain barrier (BBB) and blood–tumor barrier.^9^, ^12^, ^13^, ^14^ Hence, delivery systems should be developed to release genetic tools at the tumor site, enhancing their accumulation in cancerous cells and strengthening their effects against cancer cells.

**FIGURE 1:**
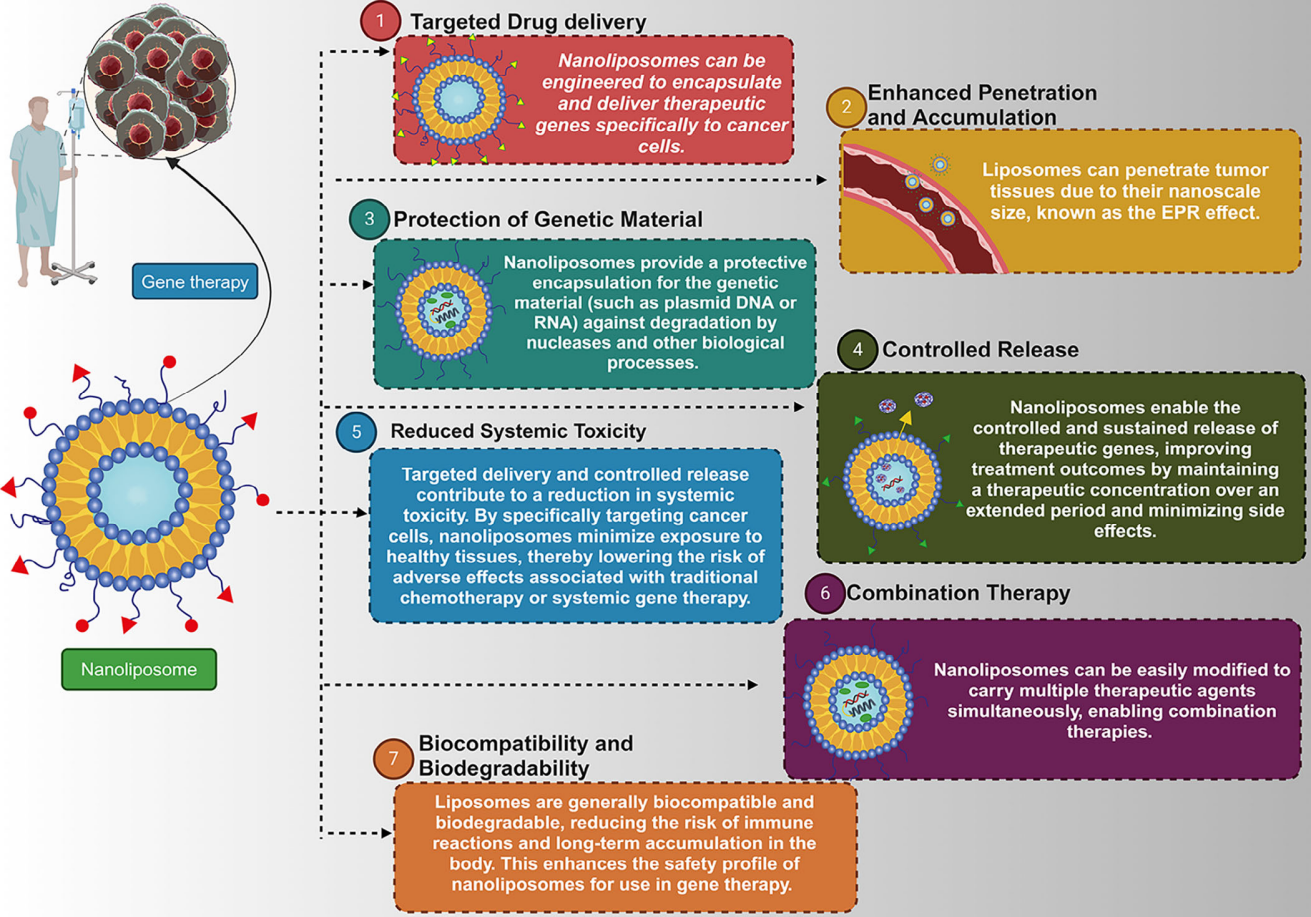
Advantages of nanoliposomes in gene therapy.

The two primary categories for delivering genetic tools are viral and nonviral carriers. Special attention has been given to nonviral vectors due to their biocompatibility, safety, and lower immunogenicity compared with viral vectors.^15^ Besides, ligands can modify nonviral vectors to significantly enhance their selectivity toward cancer cells. Selecting a suitable vector is crucial for ensuring the efficacy of the treatment.^16^ Extensive research has been conducted on two types of gene carriers, namely nanoliposomes and polycationic vectors, for the purpose of conveying genetic material. Recently, nanoliposomes have emerged as a very promising contender owing to their distinct advantages over polycationic vectors. These lipid‐based vesicles, which are very small in size, consist of amphiphilic lipid molecules. They have a wide range of uses and are commonly employed for administering drugs and gene therapy purposes. Nanoliposomes have several advantages compared with polycationic vectors in the context of gene delivery. Their capacity to interact well with biological systems, lower likelihood of causing an immune response, ability to remain stable, flexibility in modifying their surface, and ability to be produced on a large scale make them appealing. Diverse nonviral vectors, such as dendrimers and nanospheres, have been utilized in gene therapy.^17^ Nevertheless, nanoliposomes, specifically, present a hopeful framework for cancer gene therapy, offering a combination of targeted delivery, adaptable cargo, and regulated release. These distinctive benefits have garnered significant interest in the field (Figure 1).

In conclusion, the present review emphasizes the potential of liposomes as nonviral vectors for cancer gene therapy. Through a comprehensive analysis, we aim to explore the role of liposomes in delivering genetic tools, discuss their methods of synthesis and biomedical application, review strategies for introducing them to the market, and highlight their promising prospects in advancing cancer gene therapy.

## 2 LIPOSOME: BASICS, SYNTHESIS, AND BIOMEDICAL APPLICATIONS

Alec Bangham made the first identification of liposomes in 1965. Much effort has been made to implement their use for cancer therapy.^18^ There are two major categories of liposomes small and large unilamellar vesicles with particle size at the range of 100−250 nm.^19^ Among these, small unilamellar vesicles (SUVs) are those mainly applied for drug delivery.^20^ Phospholipid molecules such as cholesterol and other helper lipids are utilized for liposome synthesis.^21^ Lipophilic fatty acid chains form the hydrophobic tail of phospholipid molecules, while hydrophilic phosphate ester groups form the polar head. A number of lipid molecules can be utilized for liposome synthesis that cationic, anionic, zwitterionic and poly (ethylene glycol) (PEG)ylated lipid are among them with the capacity of being esterified to generate various phosphoglycerides.^22^ In the formation of liposomes, cholesterol is preferred because it provides lipid membrane fluidity that is important for the stability of liposomal carriers. Furthermore, in order to improve functionality, phosphate groups of small lipid molecules can be subjected to modifications.^23^ Usually liposomes consist of one or more double‐layered phospholipid vesicles (hydrophobic nature) surrounding an aqueous core.^24^ They are ideal candidates for drug delivery due to increased absorption, enhanced half‐life, reduced metabolism, and because they are minimally toxic to normal cells.^25^ The liposome properties depend on size, charge, lipid composition, and synthesis method. Some liposome features that have improved their biomedical application include biocompatibility, stability, controlled release, and biodegradability.^26^ Furthermore, surface modification of liposomes enables to selectively target certain cells, especially cancer cells.^27^

### 2.1 Synthesis

To date, various methods have been applied to synthesize liposomes. It is worth mentioning that for the various types of liposomes, such as SUVs, multilamellar vesicles (MLVs) and giant unilamellar vesicles (GUVs), different strategies have been applied for their synthesis. Electroformation, gel‐assisted formation, and emulsion phase transfer are the methods used for GUV synthesis. The solvent injection is utilized for SUV synthesis, whereas microfluidics can be used to prepare SUVs and GUVs. Film rehydration and solid rehydration are applied to synthesize SUVs and MLVs. These methods determine liposome characteristics like size, yield, type, and polydispersity. For example, liposomes prepared using film rehydration, solid rehydration, and solvent injection display large polydispersity. At the same time, electroformation, gel‐assisted hydration, emulsion phase transfer, and microfluidics generate liposomes with a low polydispersity.^28^ One of the most important aspects of liposome preparation is that vesicle formation should occur at temperatures above the *T*_m_ of the utilized lipid. This is a major issue when synthesizing liposomes as it might affect the encapsulation of components and macromolecules sensitive to temperature. For example, proteins and DNA (deoxyribonucleic acid) are sensitive to high temperatures and may lose their function.^29^ Various strategies can be applied when preparing liposomes; these can be subdivided into two major classes: solvent‐free and solvent‐displacement. In solvent‐free methods, no organic compound is used for vesicle synthesis, and amphiphiles undergo hydration in an aqueous medium. In contrast, in the solvent displacement method, the organic solvent is utilized for dissolving amphiphiles and is then removed by placing them in an aqueous medium.^30^, ^31^ Film rehydration, solid rehydration, electroformation, and gel‐assisted hydration are solvent‐free methods, while solvent injection, emulsion phase transfer, and microfluidics are solvent displacement methods.^28^

### 2.2 Biomedical applications

Noteworthy, liposomes can be utilized for the treatment of various conditions. For example, in the context of diabetes mellitus, liposomes are applied for insulin peptide delivery.^32^ Liposomes can enter macrophages and significantly diminish the secretion of proinflammatory factors, leading to the amelioration of rheumatoid arthritis.^33^ Liposomes can deliver anti‐inflammatory mediators like interleukin‐10 (IL‐10) for treatment of atherosclerosis.^34^ Liposomes are also effective in treating neurological disorders due to their ability to cross BBB; further, modification of their surface using transferrin (Tf) promotes their efficacy.^35^ Liposomes are also widely applied in treating cancer,^36^ to favor drug delivery to cancer cells. Besides, liposomes can increase the efficacy of chemotherapy agents in cancer therapy by providing targeted delivery at the tumor site.^37^ The preclinical examinations have highlighted the role of liposomal nanocarriers in the cancer suppression.^38^, ^39^ Of note, the biomedical application of liposomes is not limited to preclinical studies, as several liposomal drugs have been approved for clinical application. These include DaunoXome, for treating sarcoma; NX211 ovarian cancer therapy; Platar for solid tumors therapy, and so on.^40^ Further attempts should be made to produce liposomal carriers containing genetic tools to be used in the treatment of cancer patients. Synthesis methods and various biomedical applications of nanoliposomes are given in Table 1.

**TABLE 1:** Synthesis methods of nanoliposomes and various biomedical applications.

Method	Liposomes	Biomedical applications	References
Freeze drying (lyophilization)	MLVs, LUVs SMVs, SUVs	Vaccine delivery, drug delivery	^41^
Detergent removal (depletion)	MLVs, LUVs	In vitro biomembrane studies	^42^
Solvent (ethanol/ether) injection	SMVs, SUVs	Gene delivery	^43^
Reverse‐phase evaporation	MLVs, LUVs	Cancer therapy, imaging agents	^44^
Bangham method	MLVs,	Drug delivery, diagnosis	^45^
Microfluidic (micro hydrodynamic focusing—MHF)	SUVs, LUVs	Imaging agents	^46^
Membrane contactor	MLVs,	Drug delivery	^47^

Abbreviations: LUVs, large unilamellar vesicles; SMVs, small multilamellar vesicles.

## 3 LIPOSOMES AND GENE DELIVERY

The application of nanoliposomes in cancer therapy has significantly progressed by incorporating various nucleic acid molecules, including short interfering RNA (siRNA), short hairpin RNA (shRNA), microRNA (miRNA), long noncoding RNA (lncRNA), and CRISPR/Cas9. Due to their unique properties, nanoliposomes serve as effective carriers for these nucleic acids, facilitating targeted and controlled delivery to cancer cells. siRNA and shRNA are utilized to silence specific genes associated with cancer, while miRNA regulates gene expression at the posttranscriptional level. lncRNA functions as a modulator of various cellular processes, providing additional layers of control. The revolutionary CRISPR/Cas9 system, used for precise gene editing, is also incorporated into nanoliposomes to target specific cancer‐related mutations. This comprehensive approach, harnessing the potential of diverse nucleic acid modalities with nanoliposomal carriers, offers versatility. This section will discuss genetic tools used in cancer gene therapy.

### 3.1 DNA and mRNA delivery

The effective and specific delivery of therapeutic genetic material is a critical component of the rapidly developing field of cancer gene therapy. Liposomes, which are tiny vesicles made of lipids, have emerged as a promising method for transporting DNA and mRNA. They provide a versatile platform for precise and controlled release within cancer cells. This section explores the role of liposomes in transporting DNA and mRNA in cancer gene therapy, highlighting their mechanisms, benefits, and current research trends. DNA and mRNA can be stabilized and protected from nuclease degradation by complexing them with positively charged lipids, which enables their delivery to target cells. The delivery process involves the adsorption of liposomes onto the cell surface, followed by endocytosis and release into the cell.^48^ They offer several advantages, including encapsulation and protection of the DNA, improved cellular uptake, and targeted delivery. These benefits make liposomes an attractive option for researchers and clinicians seeking to develop effective and safe gene therapies for cancer treatment. Liposomes can be customized to improve surface properties and increase cellular uptake as drug delivery systems. This is particularly crucial in cancer gene therapy, where the efficient delivery of therapeutic DNA into cancer cells is essential for treatment efficacy. By modifying the surface properties of liposomes, researchers can optimize their ability to penetrate the cell membrane and deliver therapeutic agents directly to the target cells. The encapsulation method is a promising technique that can be used to protect DNA from enzymatic degradation and immune system recognition. Encapsulating DNA within liposomes allows the therapeutic DNA to be transported to target cells with greater stability.

This method ensures that the therapeutic DNA remains intact during its journey to the target cells, thereby increasing the efficacy of the treatment.^49^ Significant advancements have been achieved in the fight against cancer using messenger RNA (mRNA) cancer vaccines. These vaccines employ mRNA to instruct cells to generate particular proteins associated with cancer, triggering a strong immune reaction against tumors. The mRNA‐based vaccination stimulates an immune response against these specific targets by encoding antigens associated with tumor or cancer cells. The COVID‐19 pandemic has expedited the advancement of mRNA technology through the swift manufacturing and approval of mRNA‐based COVID‐19 vaccinations. This momentum has positively impacted the progress of mRNA cancer vaccine research. Liposomal nanoparticles are a complex and diverse platform for delivering mRNA. They offer a protective, adjustable, and targeted method. As previously stated, the lipid content of liposomes may be adjusted to enhance stability, biocompatibility, and controlled release in mRNA transportation. Therefore, it protects the mRNA from enzyme degradation and guarantees its integrity while being transported to target cells.^50^ A novel delivery system that employs the cationic peptide DP7 has been developed and modified with liposome immunoadjuvant properties. The primary objective of this system was to augment the expansion of dendritic delivery of mRNA encoding individualized neoantigens (DCs) and to enhance the activation of DCs. In preclinical studies, the subcutaneous administration of mRNA complexes encoding the neoantigen significantly stimulated the production of antigen‐specific lymphocyte reactions.^51^ To summarize, liposomes constitute a diverse and promising platform for the transportation of DNA and mRNA in cancer gene therapy. Because of their capacity to encapsulate, shield, and transport genetic information to specific cells with improved specificity, they are regarded as crucial instruments for more efficient and individualized cancer therapies. The advantages of modifications of liposomes are given in Table 2.The combination of liposomal technology with cancer gene therapy has the potential to greatly impact cancer treatment by providing new opportunities for therapeutic intervention at the genetic level.

**TABLE 2:** Advantages of chemical modification of liposomes.

Chemical modification	Function	Advantages	References
PEGylation	PEGylation provides steric hindrance around nanoliposomes, protecting them from degradation in biological fluids. The presence of PEG chains on the surface of nanoliposomes prevents their recognition and uptake by immune cells, thus reducing their immunogenicity. It increases the hydrophilicity of nanoliposomes, leading to improved solubility and reduced aggregation in biological fluids.	PEGylation prolongs nanoliposome circulation time, enhancing therapeutic efficacy by reducing clearance by the reticuloendothelial system and allowing sustained delivery of RNA payloads to target tissues. Minimizes off‐target effects and improves safety profile of nanoliposomal formulations. PEGylated nanoliposomes exhibit increased accumulation in tumor tissues via the enhanced permeability and retention (EPR) effect.	^52^
Surface charge modification	Modifying the surface charge of nanoliposomes changes their electrostatic properties, which can impact how they interact with cells and tissues. Researchers can improve the binding affinity and uptake of nanoliposomes by adjusting their surface charge, resulting in better delivery of RNA payloads to target cells.	Nanoliposomes that have a positive charge can interact with cell membranes that are negatively charged in a more effective manner. This interaction results in an improved internalization and delivery of RNA payloads into the cells. Nanoliposomes can be designed with improved targeting specificity for specific cell types or disease sites by regulating the surface charge, thus reducing off‐target effects. Modifying the surface charge of nanoliposomes can enhance their stability in biological surroundings by mitigating aggregation and opsonization.	^53^
Ligand conjugation	Conjugation of ligands enables the selective targeting of nanoliposomes toward cells or tissues that express corresponding receptors. Integration of targeting ligands onto the surface of nanoliposomes can augment their binding affinity and internalization by the target cells, thereby resulting in better transportation of RNA payloads.	Ligand conjugation allows nanoliposomes to selectively target cells expressing specific receptors. Delivery of RNA payloads is minimized with targeted nanoliposomes, reducing toxicity and side effects. Conjugating ligands is an approach that can be customized to aim various cell types or markers of diseases.	^54^
pH‐sensitive lipids	pH‐sensitive lipids respond to changes in pH by undergoing structural transitions, particularly in acidic environments like endosomes and lysosomes. They destabilize the liposomal membrane at low pH and promote fusion with the endosomal membrane, facilitating the release of RNA payloads into the cytoplasm for therapeutic effects.	Facilitate endosomal escape. pH‐sensitive lipids change in acidic environments, like endosomes. This controls the release of RNA payloads within target cells, minimizing off‐target effects.	^55^
Stealth lipids	Lipids that are known as stealth lipids are developed to reduce the identification by the immune system, particularly by the mononuclear phagocyte system (MPS) that includes macrophages and phagocytes. These lipids are usually made up of polyethylene glycol (PEG) chains, which create a hydrophilic polymer brush on the surface of nanoliposomes. The PEG chains produce a hydrated layer that prevents the absorption of opsonins and other serum proteins, thereby decreasing the identification and ingestion by phagocytic cells.	Nanoliposomes can evade detection by the immune system and remain in the bloodstream for a longer duration due to the presence of stealth lipids that confer stealth properties. Stealth lipids help reduce the activation of the immune system and the production of proinflammatory cytokines by minimizing recognition and uptake by phagocytic cells.	^18^
Aptamer conjugation	Aptamers have high affinity and specificity for their target molecules, making them useful for delivering nanoliposomes to diseased cells or tissues. This targeted approach minimizes off‐target effects and improves the therapeutic efficacy of RNA payloads by enhancing the accumulation of nanoliposomes at the desired site.	By conjugating aptamers, nanoliposomes can be delivered selectively, reducing the exposure of RNA payloads to the entire body. This approach helps to minimize the potential side effects and toxicity of the treatment. Utilizing aptamer conjugation is a flexible approach that can be customized to focus on a diverse set of disease markers or cell types.	^56^

### 3.2 SiRNA delivery

RNA interference (RNAi) based on siRNA enables gene silencing at the posttranscriptional level. It provides an effective degradation or translation inhibition of the target mRNA.^57^ RNAi is preferable to conventional therapeutics, including well‐known gene silencing mechanisms, due to the fast and highly selective binding and the ability to target any genes, including those encoding for undruggable protein products.^58^ Typical siRNAs have a length of 21−23 nucleotides and can be derived from long pieces of double‐stranded RNA using specific enzymes.^59^ The RNAi therapy using siRNA represents a novel form of treatment for different diseases, including cancer, viral infections, ocular diseases, and another disease in which gene dysregulation occurs.^60^, ^61^ However, some challenges are associated with siRNAs gene knockdown, including degradation by nucleolytic enzymes, immune cell uptake, and insufficient tissue penetration.^62^ Therefore, nanoscale delivery systems have been developed to optimize siRNA delivery to increase its application in the cancer therapy.^10^, ^63^ Liposomes can internalize siRNA into tumor cells via endocytosis (clathrin‐mediated endocytosis and micropinocytosis), and through cell membrane cholesterol‐dependent processes.^64^ In the next sections, we discuss how liposomes can be ideal candidates for siRNA delivery and suppressing cancer progression.

#### 3.2.1 SiRNA‐ and antitumor compound‐loaded liposomes

To date, a wide variety of antitumor compounds have been developed for cancer therapy. In this regard, nanocarriers for combined delivery of anticancer compounds and genetic tools might be considered. This section discusses using liposomes in the codelivery of siRNA and anticancer compounds in the cancer setting. The resistance of cancer cells to apoptotic cell death is mainly mediated by signal transducer and activator of transcription 3 (STAT3).^65^, ^66^, ^67^ Besides, this pathway promotes metastasis through the induction of epithelial‐to‐mesenchymal transition (EMT) and participating in the drug resistance.^68^, ^69^, ^70^, ^71^ Liposomes containing curcumin and STAT3–siRNA have been developed to treat skin cancer. These cationic liposomes represent a noninvasive topical iontophoretic method. These liposomes enter the cancer cells through clathrin‐mediated endocytosis. Upon uptake, these nanocarriers induce apoptosis and significantly diminish the cancer cell growth.^72^ In another study, the role of curcumin‐ and STAT3–siRNA‐loaded liposomes was evaluated in the context of skin cancer therapy. Interestingly, compared with intratumoral administration, iontophoretic administration is beneficial in reducing tumor progression, suppressing growth in vivo and inducing apoptosis; this suggests the potential for siRNA‐ and curcumin‐loaded liposomes in the cancer treatment.^73^

Paclitaxel (PTX) is a well‐known chemotherapeutic agent that can suppress the proliferation of cancer cells by inducing cell cycle arrest.^74^, ^75^ This results in alterations of microtubules and by preventing microtubule depolarization.^76^ PTX resistance is a common phenomenon, and one potential strategy to overcome it could rely on using siRNA.^77^ PTX‐ and survivin–siRNA‐loaded cationic liposomes have been developed to target cancer stem cells for treating brain glioma. After successful penetration through the BBB, liposomes selectively targeted glioma cells and brain microvascular endothelial cells (BCECs). Noteworthy, these liposomes induced apoptosis only in glioma stem cells but not in BCECs, showing their high biocompatibility. In glioma tumor‐bearing mice, administration of siRNA‐ and PTX‐loaded liposomes ameliorates survival. This experiment shows that siRNA‐induced downregulation of the survivin gene, a prosurvival mechanism, sensitizes cancer cells to PTX‐mediated cell death.^78^

**FIGURE 2:**
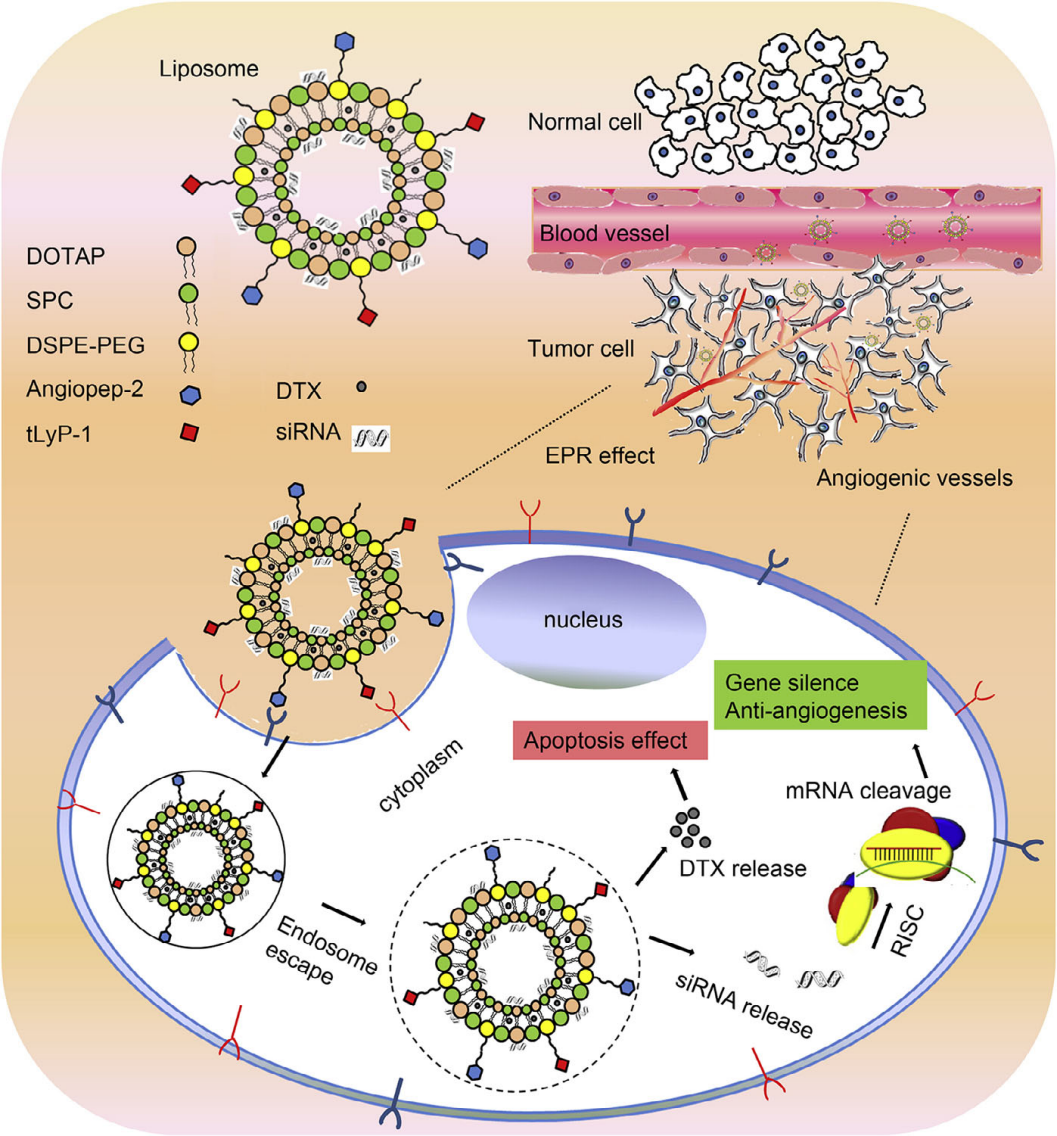
The application of liposomal nanostructures in siRNA and DTX delivery for cancer suppression. The conjugation of liposomes with Angiopep‐2 and tLyP‐1 favors their selectivity toward cancer cells. Liposomes penetrate into the cells through endocytosis and provide an endosomal escape. The increased intracellular delivery of DTX and siRNA–VEGF suppresses cancer growth and angiogenesis, therefore representing a promising tool for contrasting cancer cells. siRNA, small interfering RNA; DTX, docetaxel; EPR, enhanced permeability and retention; mRNA, messenger RNA; SPC, soybean phosphatidylcholine; DOTAP, 1,2‐dioleoyl‐3‐trimethylammonium‐propane; VEGF, vascular endothelial growth factor. Reproduced with permission from Ref. 86, Copyright (2014), Elsevier.

The tumor microenvironment (TME) is a complex space containing different types of cells, such as fibroblasts, cancer stem cells, and endothelial and immune cells.^79^ The competition among cancer cells for proliferation results in hypoxia. Increasing evidence has demonstrated the role of hypoxia in inducing chemoresistance.^5^, ^80^, ^81^ Targeting glyceraldehyde‐3‐phosphate dehydrogenase (GAPDH) might be important in cancer therapy due to a decrease in ATP levels and inhibition of autophagy. For this purpose, GAPDH–siRNA‐ and PTX‐loaded liposomes have been designed. The liposome preparation was performed using a new strategy known as cryogenic inner–outer dual reverse phase emulsion. The preclinical experiments have approved the potential of liposomes for downregulating GAPDH and enhancing the efficacy of PTX in cancer chemotherapy. Importantly, this strategy overcomes the hypoxia‐mediated drug resistance in cancer cells like HeLa and MCF‐7.^82^ A similar strategy was adopted to deliver docetaxel (DTX) for the cancer therapy. Since angiogenesis induction favors cancer progression through the vascular endothelial growth factor (VEGF),^83^, ^84^, ^85^ work has been done to develop VEGF–siRNA‐ and DTX‐loaded liposomes. Due to surface modification of liposomes with Angiopep‐2 and tLyP‐1, liposomes could penetrate the cancer cells through receptor‐mediated endocytosis, leading to enhanced intracellular accumulation of siRNA and DTX. Furthermore, the liposome structure provides siRNA and DTX escape from endosomes and lysosomes. Additional investigations revealed that VEGF–siRNA‐ and DTX‐loaded liposomes effectively induce apoptosis and suppress angiogenesis in glioblastoma therapy. Notably, no immune reaction is observed, confirming the high biocompatibility of these liposomal nanocarriers. The combination of two receptor‐specific peptides, Angiopep‐2 and tLyP‐1, mediated the liposome, as shown in Figure 2. Gene silencing of VEGF, antiangiogenesis, and apoptosis of tumor cells cooccurred in this process.^86^

#### 3.2.2 Surface‐modified liposomes

Surface modification of liposomes with different ligands and polymers is considered a vital strategy in cancer therapy since this method can significantly promote the selectivity of liposomes toward cancer cells. Therefore, it is paramount to identify which receptors on cancer cells can be targeted and which agents can be utilized for the surface modification of liposomes. This section will describe surface‐modified liposomes for siRNA delivery in cancer treatment.

Cancer progression involves different cells within the body, including TME. Th17 cells, a type of cell, produce a substance called IL‐17, which facilitates the metastasis of gastric cancer cells. Recent studies have revealed that the Tetraspanin 1 (TSPAN1) protein plays a role in activating CD4+ cells into Th17 cells. A treatment for stomach cancer has been devised that consists of a liposome polycation–DNA complex loaded with TSPAN1–siRNA and modified with Th17 antibodies. By impeding the conversion of CD4+ cells to Th17 cells, this approach actively impedes the progression of stomach cancers.^87^ The preparation of liposomes for siRNA delivery has shown potential with the use of DC‐Chol cationic lipids and DOPE neutral lipids. However, the use of cationic liposomes has several limitations, including their interaction with negatively charged plasma cells such as blood cells and serum proteins.^88^ This interaction leads to their rapid clearance and elimination from the circulation, which hinders their therapeutic application. Moreover, cationic liposomes tend to aggregate, which further complicates their use in siRNA delivery.^89^

To overcome these challenges, PEGylation has been used to enhance the stability of liposomes in serum, prevent aggregation, and improve their circulation in the bloodstream. The PEGylated cationic liposomes have been shown to enhance the efficacy of siRNA in downregulating the expression of kinesin spindle protein to suppress tumor growth in ovarian tumor therapy. The PEGylation also reduces the absorption and metabolism by the liver, while increasing accumulation at the tumor site. This has been attributed to the reduced blood clearance and promoted escape from immune recognition.^90^ It is preferable to form a complex between siRNA and polyethyleneimine (PEI), which has a positive charge. This complex formation favors the stability and ability of the liposomes to interact. The PEGylated liposomes have been used in cervical tumor therapy, where they have been shown to increase siRNA accumulation in cancer cells and reduce levels of human papillomavirus (HPV) gene. Modification with AG86 targeting peptide–amphiphile binding to α6β4 integrin has also been included to enhance selectivity toward cancer cells.^91^ These studies suggest that liposome PEGylation is a promising strategy for siRNA delivery in cancer treatment. The PEGylation approach has been shown to enhance the stability of liposomes, reduce blood clearance, and improve their accumulation at the tumor site. Furthermore, the use of AG86 targeting peptide–amphiphile binding to α6β4 integrin enhances their selectivity toward cancer cells. Therefore, the application of PEGylated liposomes in siRNA delivery could be a potent therapeutic strategy for cancer treatment.

Although some studies have reported that using PEGylation on liposomes has positive effects, there are also concerns. These concerns are serious enough to consider alternatives to PEG. When PEGylated nanoparticles are used, they might stop nanoparticles from interacting with cells, reduce cellular internalization, trigger IgM antibody production, cause accelerated blood clearance, and activate complement systems.^92^, ^93^, ^94^, ^95^, ^96^ Hence, as an alternative to PEG, PEI has been suggested. PEI has a high positive charge that allows siRNA to condense through an electrostatic interaction.^97^, ^98^ Furthermore, PEI can break down endosomal vesicles through the ‘‘proton‐sponge effect.^99^ PEI‐modified liposomes were employed to deliver siRNA and PTX for the treatment of drug‐resistant tumors, and they exhibited superior penetration capabilities compared with PEGylated liposomes in 3D spheroids.

#### 3.2.3 Advanced liposomes

##### pH‐responsive liposomes

Compared with normal and healthy tissues, cancer tissues display a more acidic pH (6.5–7).^100^ Nanoparticles are extensively applied in increasing cancer site accumulation via enhanced permeability and retention (EPR) impact.^101^, ^102^, ^103^, ^104^, ^105^, ^106^ The low pH at the tumor site derives from the rapid progression of tumors and poor blood supply.^107^ To provide specific tumor site delivery, pH‐responsive nanocarriers have been developed. pH‐sensitive nanocarriers are synthesized using various types of polymers. Cationic polymers with amino groups, anionic polymers with carboxyl groups, polymers with imidazole groups, poly (β‐amino esters), hydrazone, acetal, ortho ester, or vinyl ether‐bonded polymers, and intracellular responsive cationic polymers are some of the commonly used polymers for this purpose. Polymers with imidazole groups, polymers with hydrazine linkages, and polymers with acetal or ester linkages are among those most frequently utilized for the synthesis of pH‐sensitive nanocarriers. Noteworthy, pH‐sensitive liposomes have been designed in favor of cancer gene therapy via siRNA delivery. The selected polymer should be cleavable at the pH of the tumor site. Sometimes, these polymers favor the internalization of liposomes in the tumor cells. There are numerous advantages derived from the inclusion of these polymers into liposomes to make them pH‐responsive. Recently, siRNA delivery has been made by dioleylphosphate–diethylenetriamine conjugate (DOP–DETA)‐modified liposomes. This polymer conjugate was pH sensitive due to presence of triamine and can release siRNAs at the tumor site, enhancing their accumulation in cancer tissues. Noteworthy, it appears that this modification enhances the ability of liposomes to fuse with the membrane and mediates their penetration into cancer cells through the macropinocytosis.^108^ Based on this evidence, pH‐sensitive liposomes have several advantages, and the choice of polymer to associate is important in this regard. It has been reported that pH‐sensitive liposomes can provide synergistic effects in cancer therapy via the codelivery of antitumor compounds and siRNA.

Modifying liposomes with carboxymethyl chitosan has also resulted in the formation of pH‐sensitive nanocarriers. Loading sorafenib, an antitumor agent, and VEGF–siRNA results in a synergistic effect that induces apoptosis in hepatocellular carcinoma cells.^109^ In vivo experiments on tumor‐bearing mice show that pH‐sensitive liposomes can reach the tumor site more rapidly than other organs, exhibiting specificity toward cancer cells and tissues.^110^ The basis for developing pH‐sensitive liposomes depends on structure collapse in mildly acidic pH, releasing the cargo into the tumor cells.^111^ Combining different polymers can accelerate polymer decomposition at low pH, thus favoring siRNA release and improving the chances of success of cancer therapy. PEGylation of liposomes leads to siRNA protection against degradation and intracellular delivery of siRNA within endosomes. Embedding 1,2‐dioleoyl‐3‐dimethylammonium‐propane, a titratable lipid, into these cationic liposomes results in enhanced cationic nature of liposomes. Hence, at mildly acidic pH of TME, polymer degradation along with membrane fusion occur, this being important for the siRNA delivery.^112^ Overall, these studies indicate that liposomes can be modified with different polymers to render them pH‐sensitive; this feature enhances transfection efficiency and results in increased siRNA delivery to tumor cells and ameliorated gene knockdown efficacy.

##### Redox‐responsive liposomes

Tumor cells have a redox potential determined by oxidation and reduction levels of glutathione (GSH) and NADPH. In reducing conditions, GSH levels increase, modulating the redox microenvironment of cancer cells.^113^, ^114^ GSH controls redox levels by reducing reactive oxygen species (ROS) levels and through the formation and fragmentation of disulfide bonds.^115^, ^116^, ^117^ Various redox‐responsive nanocarriers have been developed for cancer therapy to prolong their time in circulation and provide degradation after internalization, thus facilitating the drug and gene delivery.^118^ Along with this, redox‐responsive liposomes have been developed for siRNA delivery. Although a few studies have evaluated redox‐responsive liposomes’ role in siRNA delivery, substantial work is still required to assess the potential of these liposomes in cancer therapy. Previous work was done to deploy redox‐responsive oligopeptide liposomes in mammary tumor therapy via survivin–siRNA and PTX. To develop redox‐responsive liposomes, cationic lipids (LHSSG2C_14_), natural soybean phosphatidylcholine (SPC), and cholesterol have been combined. Then, PTX was loaded in the liposome bilayer via hydrophobic interactions, while siRNA was attached to cationic liposomes through electrostatic interactions. The use of cationic lipid (LHSSG2C_14_) in these liposomal nanocarriers is advantageous because it enables the endosomal escape of nanoparticles due to the proton sponge of histidine in its structure; further, in the reducing environment of the cytoplasm, the disulfide bond in its structure is cleaved, leading to the cargo release in a redox‐responsive manner. The preclinical studies demonstrated that these redox‐responsive liposomes effectively suppress cancer growth and pulmonary metastasis due to high transfection efficiency.^119^ However, more studies are needed to modify liposomes using various polymers to make them redox‐responsive and increase siRNA delivery in cancer therapy.

##### Light‐responsive liposomes

**FIGURE 3:**
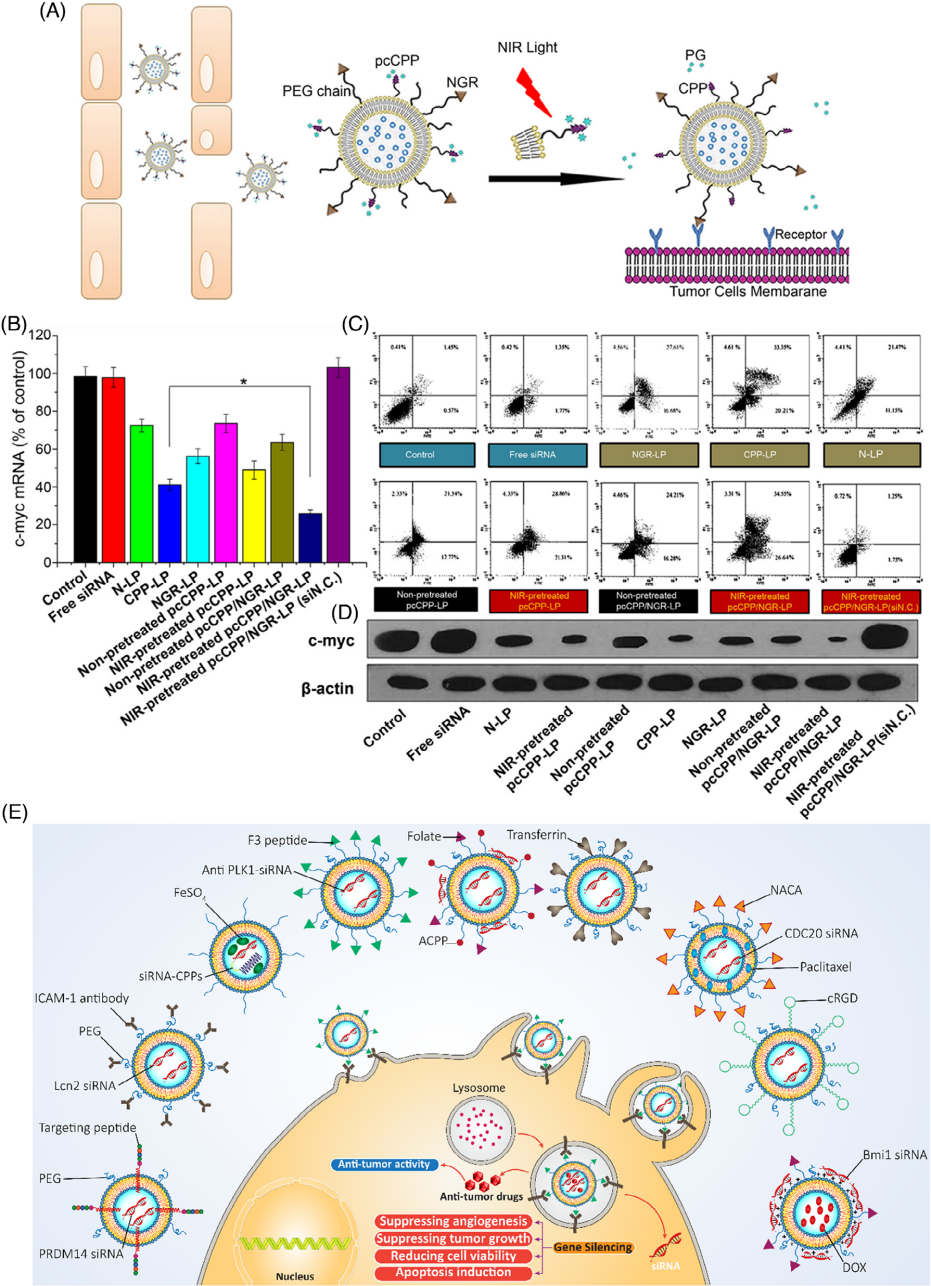
A visual representation of pcCPP/NRG‐LP and the measurement of c‐myc mRNA through qRT‐PCR. (A) Preparation of dually modified liposomes for targeted delivery at the tumor site following modification with NGR ligand. Exposure of these liposomes to NIR light results in the release of PG and subsequent interaction of CPP with the cell membrane to mediate penetration of liposomes into cancer cells; (B) application of Western blot analysis for investigating expression level of c‐myc protein. (C) The presence of apoptosis. (D) Cell apoptosis following exposure to different formulations. Reproduced with permission from Ref. 126. Copyright (2015), Elsevier. (E) Surface‐modified liposomes for the delivery of siRNA along with antitumor agents to achieve cancer suppression. This surface modification promotes penetration of liposomes into cancer cells through endocytosis. Figure was created with BioRender.com.

External stimuli like light are beneficial in cargo delivery to tumor sites for therapeutic purposes.^120^ Light is a clean and spatial stimulus that can be applied for diagnosis, cargo release, and disease therapy by modulating parameters such as wavelength, polarity, duration and irradiation intensity.^121^ Furthermore, light can induce cargo release in phototherapy, improving capacity in the disease therapy.^122^ Ultraviolet (UV) is the most common type of irradiation applied for light‐responsive nanocarriers, given the high number of polymers responsive to UV and providing enough energy for cleavage and cargo release.^123^ Such a strategy has been developed to deliver siRNA at the tumor site using light as an external stimulus.^124^ Like other kinds of smart liposomes for siRNA delivery, the polymer type is important for developing light‐responsive liposomes in cancer therapy. In a previous work, liposomes bearing photolabile‐caged peptide (PCP) were used for siRNA delivery. In this case, lysine residues on the cell‐penetrating peptide (CPP) were coated by protective groups to form PCP. After exposure to near‐infrared (NIR) irradiation, the protective groups are cleaved so that CPP re‐gains functionality. Then, CPP enhances the internalization of liposomes into cancer cells, increasing siRNA delivery at the tumor site.^125^ The application of NIR is due to damage in cells caused by UV. Figure 3A illustrates the operating mechanism of these liposomes that have been dual‐modified. Exposure to every type of liposome‐loaded c‐myc siRNA resulted in an abundance of apoptotic cells, as shown in Figures 3B and C. Cells treated with NIR‐pretreated pcCPP/NGR‐LP and loaded with c‐myc siRNA showed the highest percentage of induced apoptosis (Figure 3D). In this case, lysine residues of CPP are caged with photosensitive groups to neutralize charges. Exposure to NIR is associated with uncaging of photosensitive groups, favoring interactions of CPP with the cell membrane, and mediating internalization of siRNA into tumor cells.^126^ These advanced liposomal nanoformulations are highly relevant for tumor therapy by elevating siRNA internalization. The use of surface‐modified liposomes as delivery vehicles for siRNA and antitumor agents, which enhance cancer suppression and facilitate their internalization into cancer cells via endocytosis, is depicted in Figure 3E.^127^, ^128^, ^129^, ^130^, ^131^, ^132^, ^133^, ^134^, ^135^, ^136^ Table 3 summarizes liposomes applied for siRNA delivery in cancer treatment.

**TABLE 3:** The advantages of liposomal nanostructures in siRNA delivery.

Nanoarchitecture liposome composition	Particle size (nm)	Zeta potential (mV)	Encapsulation efficiency (%)	Surface modification/codelivery gene and/or drug	In vitro/In vivo	Cell line/animal model	Tumor	Outcomes and antitumor effects	References
siRNA‐ and phage fusion protein‐targeted liposome	–	−37.93 mV	90.5%	DMPGTVLP PRDM14	In vitro	MCF‐7 cells	Breast cancer	Fusion phage proteins provide specific targeting of breast cancer cells Downregulation of target gene in cancer cells Targeted siRNA‐protein‐liposomes were found to downregulate the PRDM14 gene by 44%.	^137^
ICAM‐1‐targeted liposomes	123 nm	−8 mV	46.7%	ICAM‐1 Lcn2	In vitro	MDA‐MB‐231 cells	Triple negative breast cancer	Lcn2 downregulation leads to VEGF inhibition Suppressing angiogenesis Suppressing tumor growth VEGF was significantly reduced by 58 ± 1% in the CM of MDA‐MB‐231 cells treated with ICAM‐Lcn2‐LP.	^138^
Thermal and magnetic dual‐responsive liposomes	90 nm	19.88 mV	87.12%	FAM	In vitro In vivo	MCF‐7 cells MCF‐7 tumor‐bearing female	Breast cancer	High cellular uptake Endosomal escape Effective gene silencing High antitumor activity in vitro and in vivo Treatment with siRNA‐CPPs/TML (activated) provided the best antitumor efficacy (228.83 ± 20.16 mm3).	^139^
F3‐targeted liposomes	–			PLK1	In vitro	PC3 cells	Prostate cancer	Reducing cell viability Downregulation of PLK1 gene at mRNA and protein levels Enhancing antitumor activity of paclitaxel against cancer cells Inducing G2/mitosis arrest PC3 and HMEC‐1 cell viability decreased by 57.0 ± 9.03% and 39.4 ± 9.86%, respectively.	^140^
PSA‐responsive PSMA‐mediated multifunctional liposomes	208.4 nm	2.79 mV		DSPE‐PEG_2000_‐ACPP, DSPE‐PEG_2000_‐CPP, PLK1	In vitro In vivo	PC3 cells Animal model	Prostate cancer	Reducing nonspecific uptake Improving prostate cancer cell recognition Downregulating PLK1 and potentiating apoptosis induction in cancer cells Providing endosomal escape Entering into cancer cells through macropinocytosis and clathrin‐mediated endocytosis Pronounced inhibition was noted in the siPLK‐1‐contained AF‐L group at both the level of mRNA (∼30% of the 5% glucose control) and protein.	^141^
Transferrin‐conjugated PEG liposomes	117.2 nm	−11 mV	92%	BCR‐ABL	In vitro	K562 cells	Leukemia	Reducing gene expression and decreasing viability of cancer cells There was an approximately 60% decrease in the expression of the BCR‐ABL gene compared with the control sample.	^142^
Cationic liposomes	100–150 nm	3–6 mV	90%	CDC20 paclitaxel	In vitro In vivo	IMR‐32 and HEK‐293 cells Neuroblastoma model	Neuroblastoma	Silencing gene Inducing cell death (80%) Apoptosis induction Delaying tumor growth in vivo Synergistic impact with paclitaxel	^143^
Carboxymethyl chitosan‐modified cationic liposomes	200 nm	−10.6 mV	90%	Sorafenib	In vitro In vivo	HepG2 cells Kunming mice bearing H22 tumor model	Hepatocellular carcinoma	The pH‐sensitive nanocarriers releasing drug and gene at pH 6.5 Sufficient delivery of siRNA to tumor site The tumors of the CMCS‐Sf‐CL group were significantly smaller in tumor volume (300 mm^3^) compared with the control groups.	^144^
cRGD grafted liposomes	118.36 nm	11.23 mV	82.93%	RRM1	In vitro In vivo	A549 cells Animal model	Lung cancer	High targeting capacity due to surface modification with cRGD Protection of siRNA against serum nucleases High stability in the presence of serum Partial hemolysis potential Reducing gene expression Suppressing cancer progression in vitro and in vivo cRGD‐CPE liposomes (2%) showed 24.2 ± 3.4% gene expression.	^145^
CPT‐PCB cationic liposomes‐Prodrug liposomes	100–200 nm	25 mV	65%	PLK1 camptothecin	In vitro	HeLa cells	Cervical cancer	Acting in a pH‐sensitive manner Rapid release of siRNA after 4 h incubation due to protonation of PCB High accumulation in nucleus Prolonged release Apoptosis induction Synergistic impact Simultaneous administration of siPlk1 with the CPT‐PCB6 system increased cell apoptosis by 63.6% with a synergistic effect.	^146^
Folate receptor‐targeted liposomes (FA‐DOX/siRNA‐L)	150 nm	43.9 mV	Up to 89.3%	Bmi1 doxorubicin	In vitro In vivo	HeLa, KB, Hep3B, A549, Huh7, MCF‐7, and LO2 cells	Different cancers	Suppressing tumor growth in vitro and in vivo Reducing Bmi1 gene expression High accumulation due to modification with folate Synergistic impact between doxorubicin and siRNA Tumors treated with FA‐DOX/siRNA‐L have the smallest sizes (248 mm^3^).	^147^

### 3.3 shRNA delivery

shRNAs are additional genetic tools applied for cancer treatment. ShRNAs belong to the family of short noncoding RNAs (ncRNAs) and downregulate gene expression similarly to siRNA. Both shRNAs and siRNAs are short ncRNAs that can be utilized for gene knockdown. In the previous section, we discussed the siRNA role and liposome delivery. Like siRNA, the use of shRNA has some caveats. For this reason, a variety of vectors have been designed for shRNA delivery; these produce short duplex RNAs in cells to then reduce gene expression.^148^ The process of gene regulation by shRNA consists of several steps. After vectors deliver shRNA to cells and following the generation of hairpin RNAs in the nucleus, cytoplasmic translocation occurs to provide shRNA cleavage by Dicer and its subsequent transformation into siRNA. Then, the target gene's expression decreases upon siRNA's embedding into RISC.^149^ From this point onward, there is a high similarity between siRNA and shRNA in gene silencing. This section aims to show how liposomes can be applied to deliver shRNA in cancer therapy.

#### 3.3.1 Enhancing shRNA efficiency

The main advantage of using liposomes in shRNA delivery is that these nanocarriers can provide long‐term silencing of target genes. Viral particles can mediate long‐term silencing via the siRNA delivery.^150^ Liposomal vectors, however, are preferable because of their safety profile. In this regard, viral vectors have been associated with inflammation, immunogenicity, mutagenesis and, more importantly, risk of oncogenic transformation.^151^, ^152^ Furthermore, shRNA effects can last for a long period.^153^ That is why studies have now focused on developing liposomes for shRNA delivery and cancer suppression. Recently, PEGylated cationic liposomes have been developed using the thin‐film hydration method. The plasmids expressing Eg5 shRNA were generated by inverted terminal repeat (ITR) sequences. This process produced Eg5 hairpin RNA, which was placed under the control of the U6 promoter. Utilizing ITRs in configuring plasmids containing Eg5 shRNA allowed the efficient expression of the desired genetic material.^154^

The next step would be the identification of molecular pathways involved in cancer survival and proliferation, followed by the administration of associated shRNA to downregulate some of these pathways. The upregulation of survivin is a common finding in different cancers and is associated with apoptosis inhibition.^155^, ^156^, ^157^ Survivin–shRNA‐loaded liposomes have been applied for the treatment of various cancers including breast, liver, melanoma, and cervical cancers. Downregulation of survivin by liposomes occurs at mRNA and protein levels, this being relevant for sensitizing cancer cells to apoptosis.^158^ Another important target in cancer therapy is thymidylate synthase (TS), an enzyme involved in tumoral DNA biosynthesis and overexpression in the ovarian cancer.^159^, ^160^ To limit peritoneal dissemination of ovarian cancer, TS‐shRNA‐loaded cationic liposomes have been applied. These loaded liposomes, containing different concentrations of TS‐shRNA (0.5, 1, and 2 mg) were injected into mice via intraperitoneal administration. Further investigations showed that combining shRNA‐loaded liposomes with PTX, an antitumor agent, enhanced the drug's antitumor effects. The interesting note is that shRNA‐loaded liposomes, administered intraperitoneally, were detected in the ascites 24 h later, therefore showing high stability in the peritoneal cavity. Furthermore, their systemic toxicity was minimized being absent in the bloodstream.^161^ The administration route is also an important aspect that impacts the liposome safety profile, as demonstrated in the case of intraperitoneal injection.

Another way to improve the siRNA delivery capacity of liposomes is to combine them with focused ultrasound (FUS). The presence of BBB diminishes efficacy in brain cancer therapy. In addition to its diagnostic applications, FUS can be utilized for opening BBB in a noninvasive and reversible manner.^162^ This is important for enhancing the efficiency of liposomes in shRNA delivery and therefore in the treatment of brain tumors.^163^ Furthermore, ultrasound‐targeted microbubble destruction significantly enhances the potential of shRNA‐loaded liposomes in cancer suppression.^164^ Overall, all these studies support the use of liposomes as carriers for shRNA delivery and cancer treatment. Various genes like Hsp70, eIF3i, and WT1 have been targeted by shRNA‐loaded liposomes and more studies are required to target additional molecular pathways responsible for cancer progression.^18^, ^165^, ^166^

#### 3.3.2 Enhancing chemotherapy efficacy

The application of genetic tools such as shRNA affects the proliferation and invasion of cancer cells and regulates their response to therapy. Inducing cell death such as apoptosis is a common and important strategy. Antitumor compounds, both synthetic and natural ones, can induce apoptosis.^167^, ^168^, ^169^ This strategy is known as chemotherapy and is considered the gold standard in cancer treatment. However, since cancer cells can activate DNA damage repair mechanisms or trigger tumor‐promoting pathways to prevent apoptosis, chemoresistance can develop.^170^ Among the different strategies applied to reverse chemoresistance, the use of shRNA has been considered, since this genetic tool can downregulate expression of genes like WT1, AMBRA1, Bcl‐xL, and PLK1 to eventually enhance the chemosensitivity of cancer cells.^171^, ^172^ The advantage of using liposomes is that they can promote gene silencing efficiency of shRNA, and simultaneously, provide a platform to mediate shRNA and anticancer compound codelivery and achieve a synergistic effect in cancer therapy. To date, a variety of experiments have applied shRNA‐loaded liposomes in cancer chemotherapy. Cationic liposomes are important tools in gene delivery due to their low immunogenicity and ease of synthesis.^173^ Their transfection efficiency, however, is low restricting their application for cancer treatment.^174^ Therefore, surface modification of liposomes with agents such as folic acid (FA) could enhance their selectivity toward cancer cells.^175^ The shRNA possesses a negative charge, and cationic liposomes can be successfully applied for their delivery. Gemini surfactants are applied to synthesize cationic liposomes because of their positive charge, presence of multivalent polar headgroups and capacity in condensing negatively charged DNA.^176^, ^177^ To enhance the efficacy of therapy against hepatocellular carcinoma, thioredoxin 1‐shRNA‐ and doxorubicin‐loaded cationic liposomes have been developed. Cationic liposomes containing gemini surfactants with symmetrical C16 aliphatic chains (L16‐2‐16) possess high cellular uptake and ability in DNA condensation. Their modification with FA promotes their penetration into cancer cells via targeting folate receptors and inducing lipid raft/caveolae‐dependent endocytosis. The shRNA/doxorubicin complex has been combined with cationic liposomes through electrostatic interactions. The complex was successful at reducing cell viability, inducing apoptosis.^178^ Besides, magnetic cationic liposomes with the capacity to codeliver STAB1–siRNA and doxorubicin have been deployed to effectively treat gastric cancer. The preclinical experiments confirmed the role of shRNA‐ and doxorubicin‐loaded liposomes in decreasing cancer growth. This codelivery approach is more effective than single delivery, promoting the synergistic impact of doxorubicin and STAB1–shRNA.^179^ In addition to doxorubicin, liposomes are able to mediate targeted delivery of DTX as another potent anticancer compound.^180^, ^181^ Furthermore, shRNAs and associated delivery systems have been also applied in combination with DTX.^182^, ^183^ In fact, the aim of using liposomes as nanostructures is to mediate targeted delivery that is of interest in enhancing the potential of shRNA in gene silencing. Such a strategy not only disrupts tumor‐promoting molecular pathways but also triggers the sensitivity of tumors to the chemotherapy.^184^ Collectively, this evidence supports the use of shRNA‐loaded liposomes to favor the cancer chemosensitivity.^185^

#### 3.3.3 Advanced liposomal nanocarriers

**FIGURE 4:**
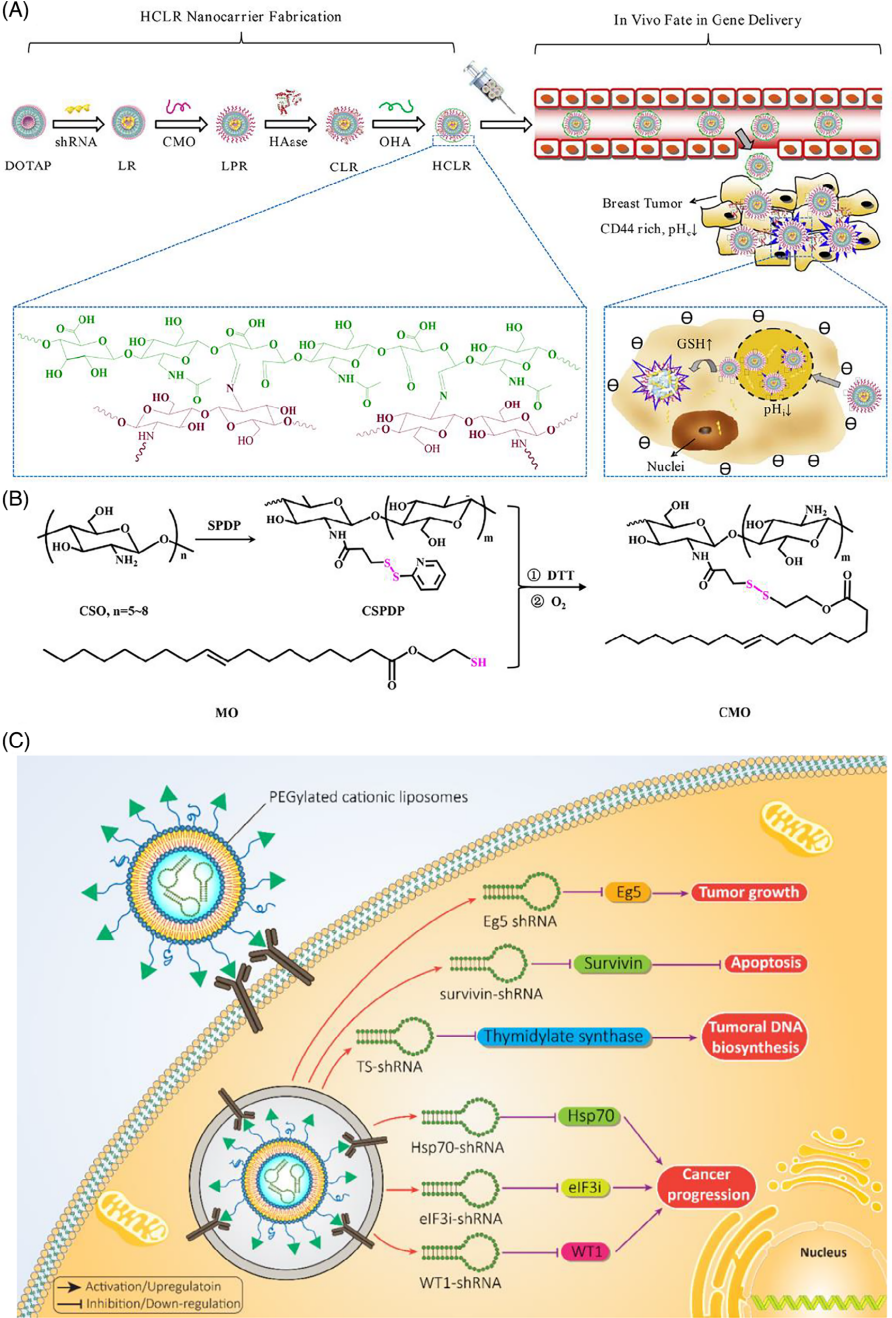
HCLR Nanocarrier fabrication and in vivo gene delivery targeting. (A) Preparation of liposomal nanocarriers that are pH‐ and redox‐sensitive for shRNA release at the tumor site. (B) Synthesis pathway. Reproduced with permission from Ref. 186, Copyright (2020), American chemical Society. (C) Similar to siRNA, the efficacy of shRNA in gene regulation can be improved using liposomes for their delivery. Surface modifications of liposomes not only improve their biocompatibility but also enhance their selectivity toward cancer cells. Upon silencing of the target gene, proliferation and metastasis of cancer cells are affected. Figure was created with BioRender.com.

In Section 3.1, we discussed different types of smart liposomes for siRNA delivery. Comparably, advanced liposomal carriers have been designed for the delivery of shRNA. To date, only pH‐ and GSH‐sensitive liposomes have been applied for shRNA delivery in cancer treatment and, in this regard, more experiments are needed. Recently, pH‐ and GSH‐sensitive liposomes containing shRNA targeting survivin have been developed for the therapy of breast cancer. Liposomes were prepared using a layer‐by‐layer method, and polysaccharides (i.e., chitosan and hyaluronic acid) were utilized for surface modification to provide redox sensitivity and favor entrance into cancer cells by endocytosis. Survivin–shRNA was then loaded into liposomes. The resulting product was HA/HAase/CS/liposome/survivin–shRNA (HCLR), as shown in Figures 4A and B. Thanks to the negative charge provided by hyaluronic acid, these liposomes demonstrated high stability in the bloodstream. Exposure to mildly acidic pH of TME (pH = 6.5–6.8), results in hyaluronic acid de‐shielding and in protonation of chitosan, confirmed with alteration of zeta potential from −23.1 to +29.9 mV. Further, the exposure of liposomal nanocarriers to 10 mM GSH induces shRNA release. By increasing cellular uptake, shRNA‐loaded liposomes successfully were able to reduce survivin expression and suppress cancer growth.^186^ These liposomes have been utilized for the codelivery of shRNA and chemotherapeutic compounds. Targeted delivery of DTX and sirtuin‐1 (SIRT1)‐shRNA by liposomes leads to a significant decrease in tumor burden. Furthermore, pH‐sensitive liposomes are more effective in breast cancer therapy compared with non‐pH‐sensitive liposomes and clinical counterparts like Taxotere. shRNA‐loaded liposomes that affect the proliferation and metastasis of cancer cells by silencing the target gene in cancer treatment are summarized in Figure 4C and Table 4.^187^

**TABLE 4:** ShRNA‐loaded liposomes in cancer therapy.

Nanoarchitecture liposome composition	Particle size (nm)	Zeta potential (mV)	Encapsulation efficiency (%)	Surface modification/codelivery Gene and/or drug	In vitro/In vivo	Cell line/animal model	Tumor	Outcomes and antitumor effects	References
PEGylated DC‐Chol/DOPE cationic liposomes	140 nm	−6.3 mV	–	pDNA‐DOPEPEGDSPE Eg5	In vitro In vivo	A2780 cells Female C57BL/6J mice	Ovarian cancer	Lack of immunogenicity Decreasing off‐targeting features of shRNA Effective targeting of cancer cells and downregulation of target gene Transfection of p_shEg5@LS caused up to 87.1% cell death, including apoptosis and necrosis.	^154^
Liposomes along with microbubbles	–	–	–	Thymidylate synthase	In vivo	SCID mice	Ovarian cancer	High stability Limited toxicity Downregulation of *TS* gene Combination with paclitaxel promotes antitumor activity and increases mice survival. It showed excellent antitumor activity of 76, 96, and 98% on days 14, 21, and 28, respectively.	^161^
Liposome combined with focused ultrasound	241.4 nm	10.9 mV	–	NGR peptide Birc5	In vitro In vivo	C6 cells Orthotopic C6 glioma	Glioma	High transfection efficiency Decreasing tumor growth and increasing survival time of tumor mice	^163^
Liposomes combined with microbubble	–	–	–	Metadherin	In vitro	MCF‐7, MCF‐10A, and T47D cell lines	Breast cancer	Effective downregulation of metadherin Subsequent decrease in proliferation and migration of cancer cells EMT inhibition Enhanced permeability of cells due to liposome combination with microbubbles	^164^
Liposomes	200 nm	15 mV	–	iRGD‐PEG_2000_‐DSPE eIF3i	In vitro In vivo	B16F10 melanoma cells Mouse model of lung metastasis	Melanoma	Inhibiting invasion and metastasis of cancer cells Suppressing lung metastasis of melanoma cells via eIF3i downregulation Treatment with R‐LP/shIF3i, which led to decreased expression of eIF3i, resulted in only 6% of Ki67 expression. Conversely, treatment with R‐LP/shNC resulted in 46% of Ki67 expression.	^165^
Folate‐targeted cationic liposomes	–	–	–	Thioredoxin 1 Doxorubicin	In vitro	Bel7402 cells	Hepatocellular carcinoma	Strong DNA condensation capacity Surface modification with folic acid enhanced cellular uptake Increasing intracellular accumulation of doxorubicin Downregulation of thioredoxin 1	^178^
Magnetic cationic liposomes	135–319.4 nm	+19.8 to +52 mV	–	SATB1 Doxorubicin	In vitro In vivo	MKN‐28 cell line Xenograft mouse model	Gastric adenocarcinoma	Suppressing growth of gastric cancer SATB1 downregulation Delivery of doxorubicin at the tumor site Synergistic impact between doxorubicin and SATB1 On day 15, the tumor volume in mice treated with TSMCL‐DOX‐shSATB1 was 0.44 ± 0.05 cm^3^.	^179^
Liposomes	110.3 nm	+18.5 mV	88.9%	PFKFB3 Docetaxel	In vitro In vivo	A549 (CCL‐ 185), H460 (HTB‐ 177) and HEK293 (CRL‐ 1573) cells A549 tumor xenograft model in nude mice	Non‐small cell lung cancer	Downregulation of PFKFB3 and subsequent induction of apoptosis and cell cycle arrest Downregulation of Cdk2 for cell cycle arrest induction Reducing survival via survivin inhibition Apoptosis induction via caspase‐3 upregulation and Bcl‐2 downregulation	^184^
Chitosan‐ and hyaluronic acid‐enveloped liposomes	105–162.3 nm	−20.2 to +33.9 mV	–	CS with a hydrophobic oleic acid tail (CMO) Hase, Survivin	In vitro In vivo	MDA‐MB‐231 cells BALB/c nude mice	Breast cancer	Smart liposomes sensitive to pH and GSH Selective targeting of cancer cells overexpressing CD44 due to surface modification with hyaluronic acid Reducing tumor growth via survivin downregulation. The silencing efficiency reached 72.5%.	^186^
pH‐sensitive liposomes	193.8 nm	18.47 mV	66.45%	SIRT1 Docetaxel	In vitro	MDA‐MB‐231 and MCF‐7 cells	Breast cancer	Effective codelivery of SIRT1‐shRNA and docetaxel Enhancing antitumor activity of docetaxel by threefold Decreasing tumor burden (78%) pH‐sensitive SIRT1 downregulation There was a significant reduction in tumor mass (∼52% reduction in tumor burden, compared with control).	^187^
Liposome microbubbles	–	–	–	Branched polyethyleneimine Survivin	In vitro	HeLa, HepG2, Ishikawa, MCF‐7, and B16‐F10 cells	Different cancers	Downregulation of survivin at mRNA and protein levels High transfection efficiency Retarding tumor growth. pSIREN/S3 with LM and USE inhibited Survivin mRNA expression by 82.35 ± 2.84.	^188^

### 3.4 MicroRNA delivery

miRNAs are key members of ncRNAs. Most experiments related to ncRNAs include miRNAs, supporting their role in different diseases. miRNAs are endogenous, short ncRNAs 18−24 nucleotides long and capable of downregulating gene expression upon binding to the 3′‐untranslated region (3′‐UTR) of mRNA. miRNAs exert their regulation at the posttranscriptional level.^189^, ^190^, ^191^, ^192^ The miRNA biogenesis process starts from the nucleus. It is followed by translocation to the cytoplasm, where different enzymes such as Drosha, Dicer, and RNA polymerase II play a significant role. miRNA gains its full function when embedded into an RNA‐induced silencing complex (RISC) and interacting with Argonaute.^193^ MiRNAs regulate biological events such as apoptosis, proliferation, metastasis, and therapy response. They are dysregulated in different diseases, including cancer, and regulating their levels is important in the cancer therapy.^76^, ^194^, ^195^

#### 3.4.1 Codelivery

**FIGURE 5:**
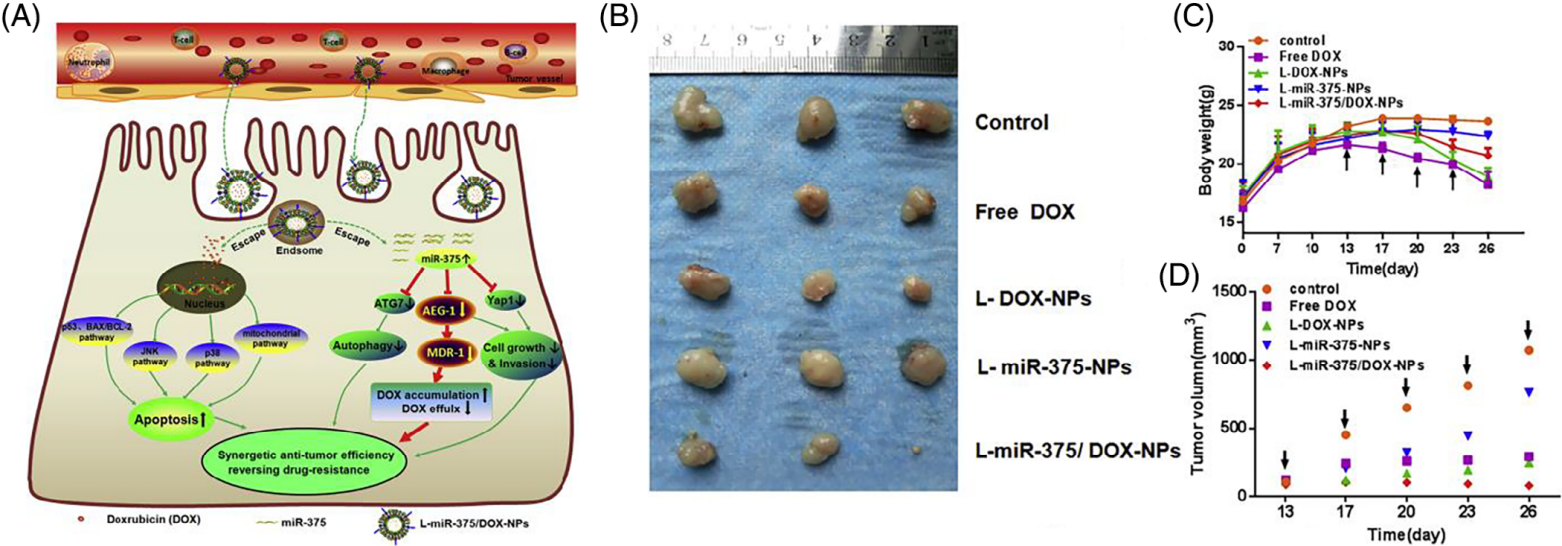
A comprehensive depiction of L‐miR‐375/DOX‐NPs that work in synergy to combat tumors and reverse the drug resistance mechanism in HCC. (A) liposomal nanocarriers in targeted delivery. The miRNA‐375 operates in the cytoplasm while DOX works in the nucleus to suppress cancer progression. (B) Tumor size. (C and D) Growth curves. Reproduced with CC‐BY license from Ref. 199, Copyright (2017), Elsevier.

As mentioned in the previous sections, chemoresistance represents an increasing challenge in treating cancer patients, and novel strategies should be applied to overcome this problem. Gene therapy using miRNAs could be considered a promising strategy in this regard. Doxorubicin has been extensively applied in the treatment of hepatocellular carcinoma, and its coadministration with icaritin enhances cancer elimination by induction of immunogenic cell death.^196^ Enhancing the expression of miRNAs is another strategy for potentiating the antitumor activity of doxorubicin.^197^ Downregulation of miRNA‐375 occurs in doxorubicin‐resistant cancer cells, and enhancing miRNA‐375 expression might help reverse drug resistance.^198^ To effectively treat hepatocellular carcinoma, miRNA‐375‐ and doxorubicin‐loaded liposomes have been developed. First, miRNA‐375 release by liposomes significantly diminishes the progression of hepatocellular carcinoma by apoptosis induction. In this case, upregulation of Bax, caspase‐3, JNK, and p38 occurs, as shown in Figure 5A. Furthermore, miRNA‐375 can stimulate cell cycle arrest (G2/M phase) and impairs cancer progression by downregulating molecular pathways such as AEG‐1, YAP1, and ATG7. Importantly, miRNA‐375‐loaded liposomes can reduce the level of multidrug resistance gene 1 (MDR1) to enhance therefore the doxorubicin sensitivity of hepatocellular carcinoma cells. Compared with the control group in gross morphology, the other groups showed apparent suppressive effects of tumor growth (Figure 5B). Examination of Figures 5C and D shows that the groups treated with L‐miR‐375/DOX‐NPs maintained permanent tumor suppression, had the smallest volume, and no significant decrease in body weight was seen among these groups.^199^

MiRNA‐101 is another tumor‐suppressor factor that diminishes myeloid cell leukemia 1 (Mcl‐1) expression and promotes chemosensitivity (doxorubicin).^200^ Codelivery of miRNA‐101 and doxorubicin using liposomes significantly enhances their intracellular accumulation in hepatocellular carcinoma cells, which is particularly relevant for antitumor activity. By entering the nucleus, doxorubicin induces apoptosis by upregulation of Bax, JNK, p387, and p53. Furthermore, miRNA‐101 released from liposomes downregulates the expression of NLK, Rab5A, Mcl‐1, EZH2, and STMN1 which are responsible for cancer malignancy. The highest inhibitory effect is observed when miRNA‐101‐ and doxorubicin‐loaded liposomes are utilized.^201^

CP is another well‐known chemotherapeutic agent applied in the cancer therapy.^202^ Because of the development of drug resistance, nanoparticles like liposomes have been developed to deliver this drug.^203^, ^204^ MiRNA‐1284 is a tumor‐suppressor factor enhancing the antitumor activity of CP via downregulation of high mobility group box 1 (HMGB1), inhibiting apoptosis and proliferation.^163^ When suppressing cervical cancer progression, codelivery of miRNA‐1284 and CP via liposomes results in significant apoptosis (60%) compared with CP (20%) or miRNA‐1284 (12%) alone. By enhancing the time in circulation and reducing clearance, liposomes effectively deliver miRNA‐1284 and CP to reduce tumor growth in vivo.^205^ No univocal plan has been developed to overcome drug resistance, each experiment offering a different strategy. Based on work already performed, codelivery of genes and chemotherapeutic agents using nanocarriers is considered the best option. In addition to their favorable biocompatibility profile and ability to enhance the accumulation of genes and antitumor agents, liposomes are applied to reverse drug resistance. In the next section, we will demonstrate how surface modification of liposomes can improve their profile as nanocarriers for gene delivery and cancer suppression.

#### 3.4.2 Surface‐modified liposomes

Liposomes can be customized to target cancer cells using specific receptors that are overexpressed on these cells. By identifying these receptors, liposomes can be modified to enhance their selectivity toward cancer cells. Tf is a blood plasma protein that is vital in transporting iron to the cells upon binding to the Tf receptor (TfR). TfR is overexpressed in hepatocellular carcinoma, making it a great target for surface modifications of liposomes.^206^, ^207^, ^208^ Chemically modified liposomes with Tf as a targeting ligand were used to deliver anti‐miRNA‐221, which inhibits liver cancer growth by increasing the expression levels of phosphatase and tensin homolog (PTEN) and tissue inhibitors of metalloproteinase 3 (TIMP3).^209^ In addition to ligand modifications, liposomes can also be modified with various polymers, such as PEG, which have been reported to improve their delivery and pharmacokinetic features.^210^, ^211^ PEGylation enhances liposomes’ systemic circulation, which increases their accumulation at the tumor site by providing an EPR effect.^142^ Recent advances in the field of targeted drug delivery have led to the development of α‐tocopherol‐based PEGylated liposomes for the delivery of miRNA‐134. The use of α‐tocopherol has been found to enhance the antitumor activity of liposomes and provide a synergistic effect by inhibiting P‐glycoprotein (P‐gp). The delivery of miRNA‐134 via liposomes results in the downregulation of Forkhead Box M1 (FOXM1), leading to a subsequent decrease in the proliferation and survival of cancer cells. Additionally, miRNA‐134‐loaded liposomes have been shown to induce apoptosis in cancer cells, up to 38%.^212^ Liposomes have the advantage of providing targeted delivery of miRNAs while displaying high biocompatibility and having minimal adverse effects on erythrocytes.^213^

**FIGURE 6:**
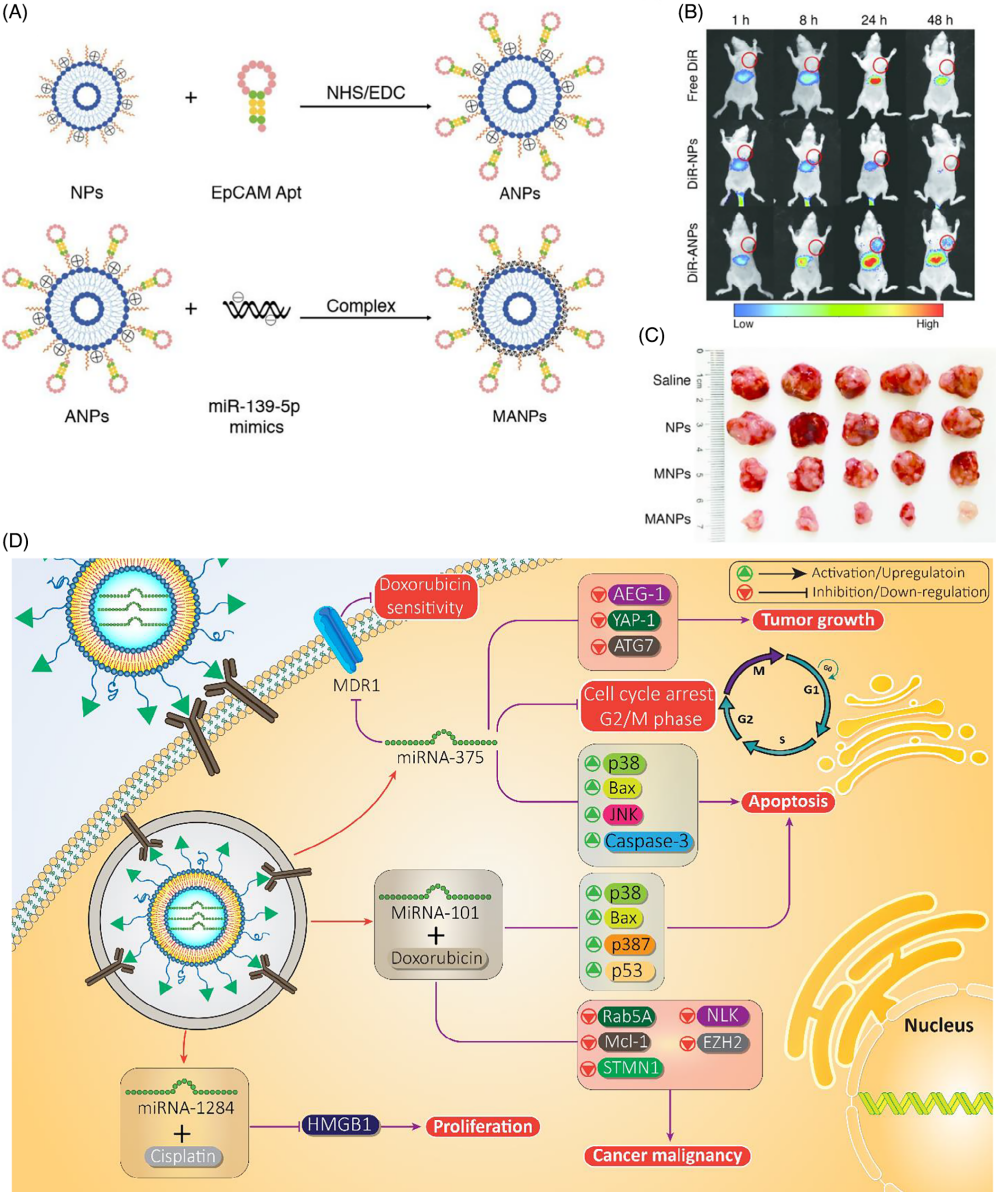
The fabrication of NPs and ANPs and in vivo targeting and biodistribution of ANPs. (A) Preparation of nanoparticles. (B) In vivo fluorescence images of xenotransplanted mice after intravenous injection of free DiR, DiR‐NPs, and DiR‐ANPs for 1, 8, 24, and 48 h. The dashed circles indicate the tumor foci in mice. (C) Tumor sizes. Reproduced with permission from Ref. 219, Copyright (2019), American Chemical Society. (D) Liposomal nanocarriers are efficient vectors for delivering miRNAs and promote cancer suppression. Figure was created with BioRender.com.

Enhancement of transfection efficiency is a crucial aspect of nanocarrier‐mediated delivery, and aptamers have demonstrated promise in this regard.^214^ Unlike conventional antibodies, aptamers possess low immunogenicity, high targeting capacity, greater stability, and can be easily synthesized.^215^ This makes them an ideal candidate for enhancing the selectivity of nanocarriers. The overexpression of epithelial cell adhesion molecule (EpCAM) is a hallmark of various cancers, while it maintains normal expression in healthy cells.^216^, ^217^, ^218^ The EpCAM aptamer can be used to target these cancer cells with higher specificity. To this end, EpCAM aptamer‐functionalized cationic liposomes have been developed for miRNA‐139‐5p delivery. These liposomes have a particle size of 15.3 nm and display a round‐shaped structure. Further investigations have revealed that these nanocarriers have a negligible hemolysis rate. Upon delivery, they effectively delivered miRNA‐139‐5p to the tumor site, leading to a decrease in the growth and invasion of colorectal cancer (Figures 6A–C).^219^

In a separate set of experiments, researchers utilized aptamer AS1411 to modify the surface of liposomes for use in ovarian cancer therapy. The results indicated that miRNA‐19b aptamer‐functionalized liposomes were effective in reducing cell proliferation and viability in this specific context.^220^ This evidence highlights liposomes as a promising candidate for miRNA delivery, which can help limit the progression of cancer. Additionally, when combined with chemotherapeutic agents, liposomes can promote cancer cell sensitivity. The incorporation of miRNAs into liposomes has also been shown to inhibit downstream targets responsible for cancer progression.^221^ The potential of miRNA‐loaded liposomes in cancer therapy is comprehensively reviewed in Table 5. Furthermore, Figure 6D offers a schematic representation of miRNA‐loaded liposomes and their mechanisms of action in cancer suppression.

**TABLE 5:** MiRNA‐loaded liposomes in cancer therapy.

Nanoarchitecture liposome composition	Particle size (nm)	Zeta potential (mV)	Encapsulation efficiency (%)	Surface modification/codelivery gene and/or drug	In vitro/In vivo	Cell line/animal model	Tumor	Outcomes and antitumor effects	References
Liposome	117.7–162.5 nm	15.3–52.4 mV	Up to 85%	MiRNA‐375 Doxorubicin	In vitro In vivo	HepG2 and SMMC‐7721 cells Xenografts		Reducing expression levels of ATG7, YAP1 and AEG‐1 Inducing apoptosis and cell cycle arrest (G2/M phase) Activating caspase cascade JNK upregulation Preventing drug resistance development via downregulation of MDR1 expression HCC cells internalized L‐miR‐375/DOX‐NPs.	^199^
Liposome	119.4–159.8 nm	17.6–43.6 mV	Up to 87%	MiRNA‐101 Doxorubicin	In vitro In vivo	Huh7, SMMC‐7721 and HepG2 cells Xenograft model of hepatocellular carcinoma		Decreasing colony formation, proliferation and invasion Inducing apoptosis Combination of chemotherapy and gene therapy The expression of Caspase‐3 was induced, and the expression of the apoptosis‐suppressing gene Bcl‐2 was suppressed by miR‐101/DOX‐L. Additionally, miR‐101/DOX‐L simultaneously stimulated the expression of Bax, which encodes a dominant inhibitor of the Bcl‐2 protein.	^201^
CD59sp‐conjugated liposomes	168.4 nm	16.2 mV	–	MiRNA‐1284 Cisplatin	In vitro	HeLa cells		Cancer elimination in a concentration‐dependent manner Effective delivery of miRNA‐1284 and subsequent downregulation of HMGB1 Prolonging blood circulation Enhancing cytotoxicity of cisplatin against cancer cells Apoptosis induction The combination of CD/LP‐miCDDP showed the highest level of apoptosis effect, reaching around 60%.	^205^
Tf‐targeted liposomes	122.5 nm	−15.74 mV	70%	MiRNA‐221 hydrogenated soybean phospholipid (HSPC)/cholesterol (CHOL)/1,2‐distearoyl‐sn‐glycero‐3‐phosphoethanolamine (DSPE)‐methoxy (polyethylene glycol) (mPEG)	In vitro In vivo	HepG2 cells Female BALB/c‐nu mice		Higher efficiency compared with nontargeted liposomes Effective delivery at the tumor site Upregulation of PTEN, P27 and TIMP3 by delivered miRNA‐221 for cancer suppression. Tf‐RL demonstrated high efficacy in silencing gene expression in HepG2 cells.	^209^
TPGS‐based PEGylated liposome	134 nm	15.1 mV	–	MiRNA‐134	In vitro In vivo	sSCC cell line Tumor mice		Increasing apoptosis (38%) Suppressing cancer cell metastasis Low growth rate Downregulation of FOXM1 by miRNA‐134	^212^
Stearylamin liposomes	70–100 nm	−1.1 mV	–	Anti‐miRNA‐191	In vitro	MCF‐7 and ZR‐75‐1 cells	Breast cancer	Partial toxicity on erythrocytes Suppressing cancer cell growth Apoptosis induction Impairing cancer metastasis Enhancing sensitivity of breast cancer cells to chemotherapeutic agents such as cisplatin and doxorubicin	^213^
EpCAM aptamer‐functionalized cationic liposomes	150.3 nm	37.8 mV	–	EpCAM Apt MiRNA‐139‐5p	In vitro In vivo	HCT116 cells Animal model of colorectal cancer	Colorectal cancer	Selective delivery of miRNA‐139‐5p to cancer cells due to surface modification with epithelial cell adhesion molecule (EpCAM) Partial blood hemolysis Suppressing proliferation and metastasis of cancer cells High cellular uptake Decreasing tumor growth in vivo The tumor volume of mice treated with MANP was only increased by 351.8 mm^3^ compared with NPs (increased by 2002.1 mm^3^)	^219^
Cationic liposome	120–600 nm	Up to 40 mV	–	MiRNA‐145	In vitro	293T cells, hepatoma cells (HepG2), cervical cancer cells (HeLa), breast adenocarcinoma cells (MCF‐7), lung adenocarcinoma cells (A549), human gastric cancer (BGC‐823) and human colorectal cancer cells (LoVo)	Different cancers	High transfection efficiency High biocompatibility (low cytotoxicity toward normal cells) Enhancing miRNA‐145 expression and subsequent downregulation of CDK6, cyclinD1, c‐Myc and Sp1 Proliferation inhibition The expression of miR‐145 resulted in the downregulation of c‐Myc mRNA expression by about 18%. Additionally, Sp1 mRNA expression was downregulated by approximately 35%.	^221^
Cationic liposomes	–	–	–	DOTAP/cho MiRNA‐101	In vitro	HeLa and Siha cells		High transfection efficiency High biocompatibility Cationic liposomes as candidates for miRNA‐101 delivery in cancer treatment	^222^

### 3.5 lncRNA delivery

**FIGURE 7:**
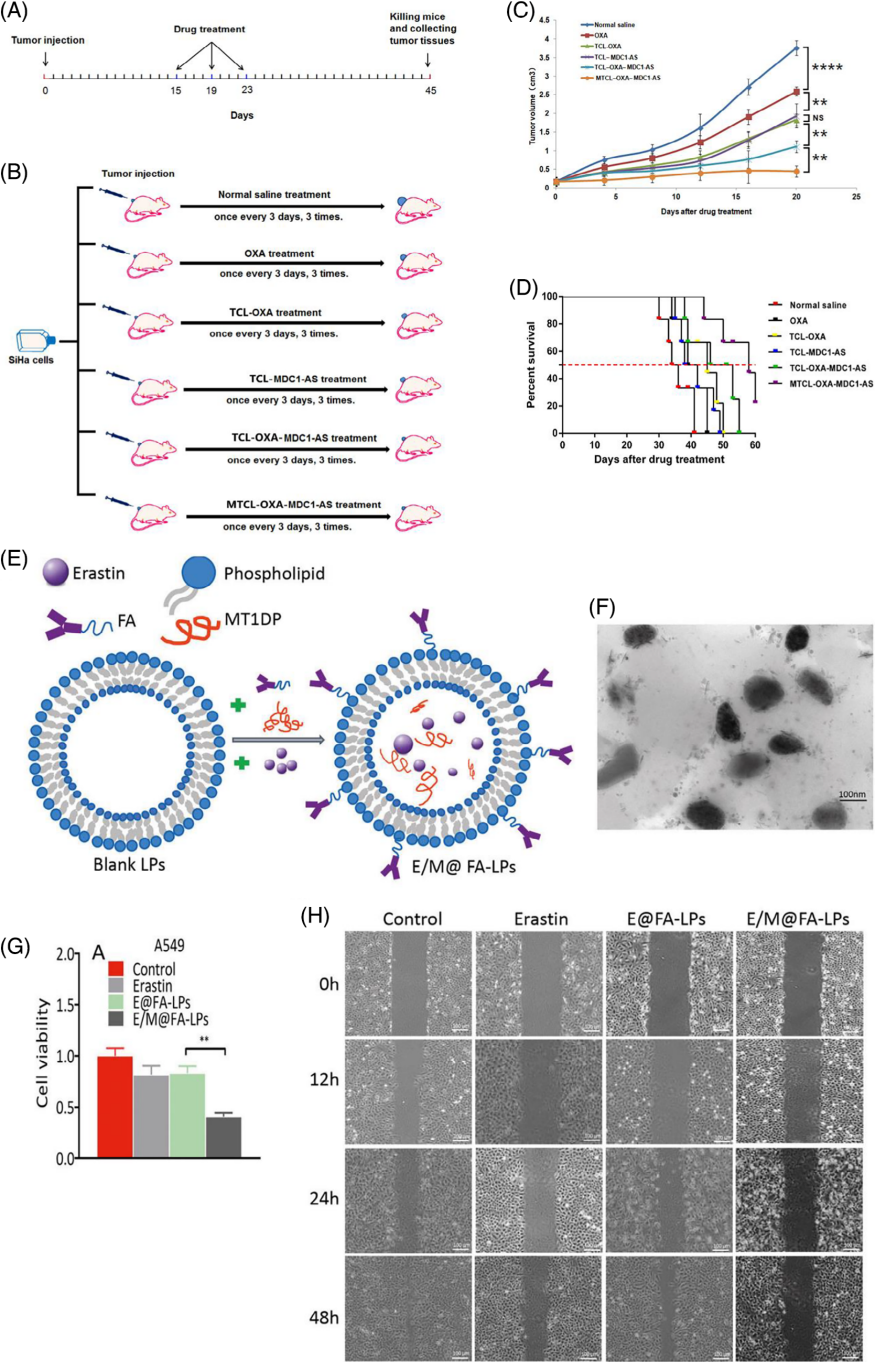
lncRNA delivery with nanoliposome for cancer gene therapy. (A) Drug administration procedure. (B) Drug administration in mice. (C and D) In vivo antitumor test (C: size of transplanted tumor, D: survival curve). **p < 0.0001. Reproduced with CC‐BY license from Ref. 235, Copyright (2017), International Journal of Nanomedicine. This is a schematic diagram of the preparation of E/M@FA‐LPs and its antitumor effect. (E) Nanoparticle synthesis. (F) TEM images. (G) MTT assay. (H) Migration investigation of tumor cells exposed to liposomes. Reproduced with CC‐BY license from Ref. 237, Copyright (2020), Nature Journal.

LdncRNAs are ncRNAs with various physiological functions in cells.^223^, ^224^, ^225^ LncRNAs are more than 200 nucleotides long and do not encode for proteins.^226^, ^227^, ^228^ RNA polymerase II transcribes lncRNAs without being translated. Increasing evidence has demonstrated that lncRNAs regulate biological events such as apoptosis, proliferation and migration, deregulated in different tumors.^11^, ^229^, ^230^ For this reason, lncRNAs can be considered diagnostic and prognostic tools in cancer setting.^231^, ^232^, ^233^, ^234^ To date, a few studies have examined the role of liposomes in lncRNA delivery and have all confirmed the potential of these nanocarriers. Recent work has shown that anti‐lncRNA‐loaded liposomes can mediate the chemosensitivity of the cervical tumor. Targeted delivery of anti‐lncRNA ediator of DNA damage checkpoint 1 via liposomes significantly diminishes the progression of the cervical tumor. This antitumor activity benefits oxaliplatin sensitivity (Figures 7A–D).^235^

The lncRNA metallothionein 1D pseudogene (MT1DP) is another factor, the expression of which undergoes downregulation in different cancers. It has been shown that enhancing the expression levels of MT1DP is correlated with a decrease in viability and colony formation of the liver tumor.^236^ Targeted delivery of lncRNA MT1DP using folate‐modified liposomes induces ferroptosis in lung tumor. In this way, MT1DP promotes the stability of miRNA‐365a‐3p to downregulate the nuclear factor erythroid 2‐related factor 2 (Nrf2). Upon inhibition of Nrf2 signaling, ROS levels increase, resulting in ferroptosis and decreased viability of lung cancer cells (Figures 7E–H).^237^ Previous work has shown that siRNA‐loaded liposomes can promote cancer elimination. Since siRNA is a promising tool in reducing the levels of lncRNAs. siRNA‐loaded liposomes have been applied to target lncRNAs in other disease settings.^238^ Therefore, this strategy can be applied in future work to target lncRNAs responsible for tumor growth. Liposomes as effective nanocarriers could enhance efficacy in gene silencing.

### 3.6 CRISPR/Cas9 delivery

The CRISPR/CRISPR‐associated protein (Cas) systems are related to the adaptive immune systems of archaea and bacteria. In recent years, considerable effort has been spent to make CRISPR applicable to the disease therapy.^239^, ^240^, ^241^ CRISPR/Cas9 is the most common type of CRISPR system that induces cleavage in double‐stranded DNA via Cas9, an endonuclease guided by crRNA.^242^ Upon DNA cleavage, molecular mechanisms responsible for genome editing, such as nonhomologous end joining (NHEJ) and homology‐directed repair pathways, are activated.^243^ These mechanisms can lead to end joining, base insertion and deletion or directional mutation using a homologous repair template.^244^, ^245^, ^246^ Recently, the CRISPR/Cas9 system has been applied to treat various tumor kinds, including mammary, cervical, ovarian, and lung cancers, by targeting signaling networks. In addition to reducing tumor progression, CRISPR/Cas9 system can be applied to elevate drug sensitivity. However, this system suffers from off‐targeting effects that could be overcome using vectors.^247^, ^248^, ^249^, ^250^, ^251^, ^252^, ^253^ In this section, we discuss the use of liposomes for the targeted delivery of the CRISPR/Cas9 system in cancer treatment.

As mentioned earlier, the CRISPR/Cas9 system is a novel strategy in gene regulation, and because of its ease of use and synthesis, it has been increasingly applied to multiple settings. Off‐targeting effects and degradation by enzymes are two important issues limiting its efficiency in gene silencing. It has been reported that encapsulation of the CRISPR/Cas9 system by liposomes protects it from DNase I degradation while providing targeted delivery. Furthermore, liposomes enable controlled release of the CRISPR/Cas9 system, which is important when increasing gene regulation efficiency.^254^ In previous sections, we have discussed the development of advanced liposomes for the gene delivery.^255^ Most liposomes are designed based on their response to internal stimuli, the pH being the most important one. To enhance targeted delivery of CRISPR/Cas9, pH‐sensitive cationic liposomes have been generated to effectively downregulate HR‐HPV16E6/E7 oncogene, leading to apoptosis induction and reducing the proliferation of cervical cancer cells. These liposomes display prolonged time in circulation and display high biocompatibility. Furthermore, intratumoral injection into nude mice significantly decreases tumor growth.^256^ For all these reasons, they are promising candidates for CRISPR/Cas9 delivery in cervical cancer treatment.

**FIGURE 8:**
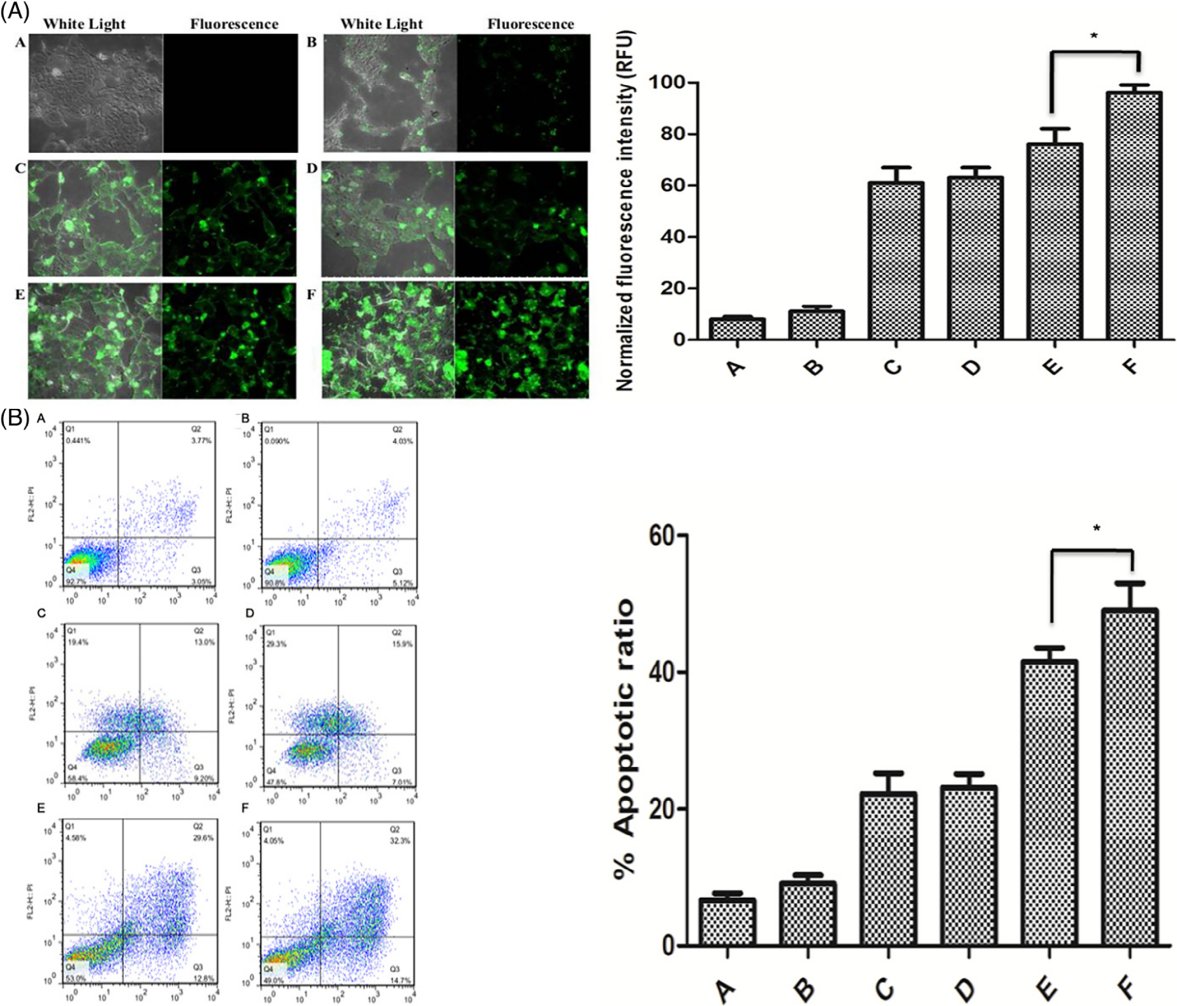
Liposomes for CRISPR/Cas9 delivery and cancer treatment. (A) Exposing tumor cells to liposomes. (B) Apoptosis assay. Reproduced with CC‐BY license from,^265^ Copyright (2017), Oncotarget.

Recently, an effort has been made to use liposome‐template hydrogel nanoparticles (LHNPs) for CRISPR/Cas9 delivery. Compared with other types of nanocarriers, such as lipid nanoparticles^257^ and DNA nano clews,^258^ LHNPs demonstrate two significant advantages. The first is that LHNPs can provide CRISPR/Cas9 delivery at the protein form, leading to significant increase in efficiency and specificity.^259^ Second, LHNPs can cross the BBB and deliver the CRISPR/Cas9 system to the brain, making them suitable for the delivery of gene therapy in the setting of brain tumors.^126^, ^260^, ^261^ Due to these advantages, work has been done to use LHNPs for the delivery of CRISPR/Cas9 in glioma treatment. These nanocarriers successfully downregulate polo‐like kinase 1 (PLK1) expression to suppress cancer progression.^262^ Regarding the tumor‐promoting role of PLK1 in cancer, significant effort has been made to inhibit this factor. PLK1 activation is responsible for cancer progression and drug resistance, while its downregulation exerts antiproliferative effects.^263^, ^264^ To treat prostate cancer, aptamer‐functionalized liposomes containing CRISPR/Cas9 for PLK1 manipulation have been developed. Figure 8A shows the fluorescently tagged CRISPR/Cas9 uptake in the cytoplasm of cells. Liposome chimeras with A10 aptamer showed the most potent gene silencing effect due to the efficient delivery of CRISPR/Cas9 into LNCap cells. In Figure 8B, apoptosis was observed in LNCaph cells due to downregulation of PLK‐1. The modification of liposomes by aptamers results in the selective targeting of prostate cancer cells overexpressing prostate‐specific membrane antigen (PSMA).^265^

Another challenge in cancer therapy relates to the fact that cancer cells can stimulate molecular pathways that are responsible for reducing antitumor immunity. Programmed death‐1 (PD‐1) signaling activation results in central and peripheral T‐cell tolerance. Due to the role of PD‐1 signaling in reducing T cell proliferation and toxicity against cancer cells, inhibitors have been developed for effective cancer immunotherapy.^266^, ^267^, ^268^ The CRISPR/Cas9 system has been applied to regulate PD‐1 and ultimately enhance antitumor immunity, suppress tumor growth, and activate receptors beneficial for cancer immunotherapy.^269^, ^270^ Recently, a combination of CRISPR/Cas9‐loaded liposomes and dendritic/tumor fusion cells (FCs) has been applied in cancer therapy. The CRISPR/Cas9‐loaded liposomes led to the downregulation of PD‐1 in T cells this was followed by their activation by FCs. This combination significantly enhanced T cell proliferation, secretion of proinflammation cytokines such as interferon‐α (IFN‐α), and elimination of HepG2 cells.^271^ This study demonstrated the role of CRISPR/Cas9‐loaded liposomes in enhancing antitumor immunity via PD‐1 downregulation. More experiments are required to validate the true potential of CRISPR/Cas9‐loaded liposomes in cancer suppression and stimulating antitumor immunity (Table 6).

**TABLE 6:** CRISPR/Cas‐loaded liposomal nanocarriers in cancer therapy.

Nanoarchitecture liposome composition	Particle size (nm)	Zeta potential (mV)	Encapsulation efficiency (%)	Surface modification/codelivery gene and/or drug	In vitro/In vivo	Cell line/animal model	Tumor	Outcomes and antitumor effects	References
Cationic liposomes	100–150 nm	+36 mV	–	GFP (green fluorescent protein)	In vitro	HepG2 cells	Hepatocellular carcinoma	Prolonged release Protection against degradation by DNase I High cellular uptake	^254^
pH‐sensitive cationic liposomes	266.8–452.9 nm	44–76.21 mV	–	HPV	In vitro In vivo	SiHa, Caski and C33A cells Nude mice	Cervical cancer	Suppressing the proliferation of cervical cancer cells Apoptosis induction via HR‐HPV16E6/E7 downregulation Long circulation in blood stream pH sensitive and enhancing cellular uptake Reducing tumor growth in vivo	^256^
Liposome‐templated hydrogel nanoparticles			62.8%	PLK1	In vitro In vivo	U87 cells Tumor‐bearing mice	Glioma	Effective gene delivery and downregulation of PLK1 in glioma Suppressing tumor growth in vitro and in vivo	^262^
Aptamer‐cationic liposome	150 nm	40 mV	–	PLK1	In vitro In vivo	LNCaP and PC‐3 cells Male athymic nude mice	Prostate cancer	Selective targeting of prostate cancer cells overexpressing PSMA due to surface modification with aptamer Significant downregulation in gene expression Silencing capacity confirmed in vitro and in vivo	^265^
Liposome nanoparticle	–	–	–	–	In vitro In vivo	HepG2 cells and lung adenocarcinoma cell line A549	Hepatocellular carcinoma	To stimulate T‐cell activation in response to DC/tumor FCs and enable T‐cell mediated cancer immunotherapy, liposome‐encapsulated CRISPR/Cas9 genome editing technology proved effective in knocking out the PD‐1 gene in T‐cells.	^267^
Stealth liposomes	–	–	–	E7	In vitro In vivo	CasKi (HPV 16 +ve, passage number 6), C33A (HPV −ve, passage 12), HeLa (passage number 23), and Jurkat cell lines Immunocompetent C57BL/6J mice	Cervical cancer	Lack of toxicity on spleen and liver Downregulation of E7 by CRISPR‐loaded liposomes does not result in immunogenic cell death	^272^

## 4 LIPOSOME RELEASE KINETICS

Conducting in vitro release kinetics studies is imperative to better understand the release profile of RNA payloads from liposome‐based gene carriers over time. These studies provide critical insights into the kinetics of RNA release, including both the rate and extent of release. Such information is essential in optimizing the design and performance of liposome‐mediated RNA delivery systems.^273^ To this end, in vitro‐release kinetics studies are critical to developing effective gene delivery platforms.^274^ There are many factors that affect gene release behavior in nanoliposomes.^275^ The kinetics of gene release in liposomal carriers are significantly influenced by the lipid composition of the carrier. The physicochemical properties of the different lipids present in the carrier can substantially alter the permeability and stability of the liposomal membrane, thereby impacting the rate and mechanism of gene release. Hence, it is imperative to carefully select lipids with appropriate physicochemical properties to optimize gene release kinetics in liposomal carriers.^18^ The encapsulation of genes within liposomes can be influenced by the method employed, which can subsequently impact the release kinetics. Techniques such as lipid film hydration, sonication, or extrusion can give rise to variations in cargo loading efficiency and release behavior. It is important to note that the choice of encapsulation method must be made with careful consideration of the desired release profile, as well as the stability and integrity of the encapsulated material.^54^ Surface modifications, such as PEGylation or ligand conjugation, have been shown to impact gene release kinetics and the interaction of liposomes with biological environments. Stealth coatings can increase circulation time and alter release profiles while targeting ligands can enhance specificity and direct release to specific sites. pH‐sensitive lipids incorporated into liposomal membranes enable triggered release in response to changes in pH, such as those encountered in endosomes or lysosomes. The pH‐dependent release mechanisms play a crucial role in enhancing the cytoplasmic delivery of genes. The ability to modulate the interaction of liposomes with biological environments enables researchers and clinicians to tailor drug delivery systems to meet specific therapeutic needs.^276^

Yang et al.^277^ developed long‐circulating and cationic liposomes as a delivery system for siRNA to improve its cellular uptake and inhibitory activity on the expression of VEGF in cancer cells. The efficacy of the cationic liposomes was evaluated by fluorescence‐activated cell sorting studies and confocal laser scanning images, which demonstrated the increased uptake of fluorescence‐labeled siRNA in cancer cells. These findings suggest that cationic liposomes may serve as a promising platform for the targeted delivery of siRNA in cancer therapy.^277^

Yang et al.^139^ have created a new siRNA targeting system that merges the qualities of biological and physical siRNA targeting for use in magnetic hyperthermia‐triggered release. The system incorporates cell‐permeable peptides (CPPs) and magnetic materials. They loaded the siRNA‐CPP conjugate in thermal and magnetic dual‐sensitive liposomes and tested the release activity, gene silencing efficiency, targeted cellular uptake, in vivo targeted delivery, and in vivo antitumor activity of siRNA‐CPPs in vitro. By using magnetic liquid Fe_3_O_4_, they successfully achieved TML release of thermally triggered siRNA‐CPPs in the cell. This research demonstrates that such dual‐sensitive vesicles have great potential for delivering siRNA effectively for oncotherapy.^139^

Nahire et al.^111^ developed a system that utilizes pH‐teriggered chogenicity and content release from liposomes. The system takes advantage of the production of CO_2_ gas bubbles when liposomes are incubated in acidic pH buffers, which leads to echogenicity in the liposomes. Structural changes in the liposomes, caused by escaping gas bubbles, facilitate the release of encapsulated contents, with a potential release rate of up to 56%. The researchers further demonstrated that the system's release kinetics could be enhanced by the simultaneous application of diagnostic frequency ultrasound (1 MHz, 5 min), resulting in an 80% release rate. These findings hold the potential to advance the field of drug delivery systems, making it possible for targeted release of encapsulated contents in response to specific stimuli.^111^

The kinetics of gene release from liposomal carriers are crucial in determining the efficiency and specificity of gene delivery for therapeutic purposes. A thorough understanding of the factors influencing release behavior and the use of appropriate experimental techniques can facilitate the design of optimized liposomal formulations that enable precise and effective gene delivery. To summarize, the elucidation of gene release kinetics from liposomes has immense promise for advancing gene therapy strategies and addressing challenges associated with nucleic acid delivery in biomedical research and clinical practice.

## 5 LIPOSOMES AND DETERMINING FACTORS

Although previous sections demonstrate the efficacy of liposomes in cancer gene therapy, some key factors should be considered in future work aimed at developing effective liposomal nanocarriers for cancer gene therapy. The lipid composition of liposomes affects their capacity to deliver genes. For example, it has been reported that upon injection of siRNA‐loaded cationic liposomes, lipids with amine head group, linker arm, and length of alkyl groups affect the biodistribution of siRNA.^278^ Furthermore, to induce the fusion process, aromatic molecules' conical shape and presence are important. Noteworthy, the lipid composition and the formation of the complex with genetic tools affect liposome size and zeta potential, resulting in changes in the biodistribution.^279^ Liposomes with a particle size of 5−50 nm are excreted through the urine or accumulate in the liver. However, as the size increases further, penetration into cancer cells decreases, thus limiting their cellular uptake and transfection efficiency.^280^ Hence, the impact of the DNA complex on the liposome size should be considered. In addition to the size, surface charge or zeta potential is another factor impacting the functionality of liposomes. After binding to genetic tools such as siRNA, the zeta potential of liposomes decreases due to their negative charge. Furthermore, cationic liposomes deliver genetic tools that cause immune system activation and aggregation (due to low electrostatic repulsion).^281^ Finally, the protein corona is another factor that affects liposome behavior. Following in vivo administration, several proteins are absorbed onto the surface of nanoparticles, constituting the biological profile of a certain nanocarrier.^282^ The body sees and interacts with the protein corona instead of synthetic surface of a nanocarrier. Therefore, protein corona affects the in vivo fate of a nanoparticle.^283^, ^284^ Liposomes have been reported to decrease in size after contacting the plasma. The lipid composition determines the interaction of liposomes with the plasma proteins. Liposomes containing 1,2‐dioleoyl‐3‐trimethylammoniumpropane (DOTAP) interact with vitronectin, while liposomes containing DOPE specifically interact with apolipoproteins.^285^ Such interactions greatly affect the fate of nanoparticles. For example, liposomes demonstrate high cellular uptake by hepatocytes when interacting with apolipoprotein B and E.^286^ Overall, size, zeta potential, protein corona, and lipid composition determine the biodistribution and fate of liposomes. These parameters should be carefully determined before using liposomal nanocarriers to deliver genetic tools.

Incorporating histopathology in studies concerning liposomes or modified liposomes can provide significant insights into the potential side effects and tissue responses related to their use. Histopathological analysis facilitates the examination of cellular and tissue alterations, such as inflammation, necrosis, fibrosis, and other pathological changes, which may occur following liposome administration. This approach is crucial to researchers as it provides essential information regarding the safety profile and efficacy of liposomes and modified liposomes, thus enhancing their translational potential. Histopathological findings are a crucial aspect of research on the long‐term effects of liposome exposure. These findings provide insights into potential toxicity and tissue remodeling. By correlating histopathological data with other endpoints, such as biochemical analyses and functional assessments, researchers can gain a comprehensive understanding of the safety profile and therapeutic efficacy of liposomes. This approach enables a more thorough evaluation of liposomes.

Zhu et al.^287^ utilized peptide‐based cationic liposomes to conduct histopathology tests on mice in order to investigate any potential tissue damage or inflammation. The mice were inspected three times daily, and the researchers observed no discernible histopathological alterations in kidney and spleen tissue sections following CDO14 and DOTAP injection.

Zhang et al.^288^ utilized liposome technology to target M2 macrophages in their gene therapy approach aimed at developing siRNA‐loaded (siIKKβ‐ML) liposomes that inhibit kappa B kinase β (IKKβ). Upon examining the results of their experiment, which aimed to effectively reprogram the M1 phenotype and prevent proangiogenic functions, it was observed that when compared with the control group, there were no significant abnormalities in the retinal structure for either the intravitreal injection of siIKKβ‐ML or free siIKKβ, as determined through a combination of optical coherence tomography and histopathological examination.

Quin et al.^289^ have developed liposomes that are modified with perfluorooctyl bromide (PFOB@Lipo) to facilitate the loading of oxygen and its targeted delivery to tumor sites. With this approach, they aim to make it possible to alleviate the hypoxic conditions common in tumors. Then, to inhibit tumor progression, PAR‐Lipo was applied to mediate high‐efficiency delivery of the suppressor gene pTP53. Researchers used the TUNEL assay and hematoxylin and eosin to evaluate cell apoptosis. Results indicate that the combination of apoptotic cells reached maximum levels. The modified PAR‐Lipo/pTP53 generated through this method consistently exhibited effectiveness in destroying tumor cells. Tissue histopathology analyses were conducted to compare the groups, and no significant abnormalities were observed in comparison with both the control group and other groups, revealing good biocompatibility of the therapeutic procedure. Ultimately, they found that O_2_@PL was capable of carrying oxygen to eliminate hypoxia in the tumor, thereby enhancing its antitumor ability in gene therapy.

Arora et al.^290^ used a modified liposome approach to treat Alzheimer's disease, producing ApoE2‐encoding plasmid DNA (pApoE2). The liposomes aim to a special brain‐targeted glucose transporter‐1 (glut‐1). The liposomes underwent surface functionalization through the incorporation of a glut‐1 targeting ligand mannose and a CPP. The purpose of this modification was to enhance the liposomes ability to target the brain and improve their cellular internalization. It was evaluated by histopathological analysis of tissues and compared with saline control for its biocompatibility and safety. The analysis revealed that there were no signs of abnormality in the morphology, inflammatory cell infiltration or necrosis in any of the organ tissues examined. In addition, there were no irregularly shaped nuclei or any other abnormalities observed in the brain tissues of the treated mice. The liver tissue showed no signs of ballooning or inflammation, the heart tissue showed no deterioration of muscle fibers or myofibril loss, and no signs of pulmonary fibrosis were found in the lungs. According to the study, the dual‐functionalized liposomes demonstrate safety when used in living organisms. No undesirable effects, including cell death or inflammation, were observed in tissue samples collected from animals receiving the formulations. This indicates that these liposomes hold significant potential as a delivery vehicle for gene therapy in the treatment of Alzheimer's disease.

Incorporating histopathological evaluation in preclinical and clinical studies of liposomes or modified liposomes is a crucial step in comprehensively assessing their safety profiles and understanding potential side effects. Through the elucidation of tissue‐level responses and mechanisms of action, histopathology plays a vital role in the rational design and optimization of liposomal drug delivery systems for therapeutic applications. By providing insights into the structural and functional changes associated with liposome administration, histopathological analyses contribute to a more comprehensive understanding of the safety and efficacy of these systems. Therefore, researchers and clinicians should incorporate histopathological evaluation as an integral component of their studies to optimize the therapeutic potential of liposomal drug delivery systems.

## 6 PRECLINICAL AND CLINICAL STUDIES: PROGRESS AND OBSTACLES

To date, experiments confirmed the role of liposomes in cancer gene therapy. In vitro experiments demonstrated that liposomal nanocarriers containing siRNA can diminish the expression pattern of target genes and therefore reduce cancer progression. In vivo experiments showed siRNA protection by liposomes, which provide targeted delivery at the tumor site and high cellular uptake. These features are vital for suppressing cancer growth and metastasis in vivo. Due to the progress in bioinformatics, the molecular pathways responsible for cancer progression have been identified and targeted by gene‐loaded liposomes. Furthermore, upon recognition of receptors on the cell surface, related antibodies or ligands can be utilized to enhance the selectivity of liposomes toward cancer cells. Surface modification of liposomes has been deployed in preclinical studies. Advanced liposomes sensitive to pH, light and redox have been developed to optimize the internalization of liposomes into the tumor tissue. Finally, the codelivery of antitumor compounds and genetic tools by liposomes has been developed, supporting the role of liposomes in cancer gene therapy. However, the clinical application of liposomes requires further optimization in their synthesis. The large production of liposomes represents one potential problem. There should be a novel and cost‐effective way to synthesize liposomes at the scale needed for clinical application. Another caveat is the biocompatibility of gene‐loaded liposomes. It was reported that by enhancing the dose of liposomes, there is a concomitant increase in their side effects. The siRNA‐loaded cationic liposomes demonstrate partial toxicity at low doses. However, enhancing their concentration results in pulmonary inflammation and liver injury. This is mediated by the release of proinflammatory cytokines such as TNF‐α and IL‐6 and decreased anti‐inflammatory cytokines such as IL‐10.^287^ As high doses of liposomes should be applied in the treatment of cancer patients, the question remains as to how such concentration‐dependent toxicity could be overcome. These concerns should be addressed before commercialization and clinical application. The clinical studies in Table 7 represent efforts to explore the potential of liposome‐based gene delivery systems for cancer gene therapy. These studies evaluate the safety and effectiveness of liposomal vectors in delivering therapeutic genes to tumor cells, contributing to developing new treatment strategies for cancer patients.

**TABLE 7:** Clinical trials of lipoplex‐based delivery systems for cancer treatment.

Identifier	Carrier components	gene/drug	Disease	Phase (start year)	Sponsors	Purpose
NCT03323398	Not reported	mRNA encoding human OX40L	Lymphoma	I/II (2017)	ModernaTX, Inc. (Cambridge, MA, USA)	The clinical study evaluates the safety and tolerability of escalating doses of mRNA‐2416 alone and in combination with fixed administered doses of durvalumab, as well as the objective response rate (ORR) of mRNA in participants with relapsed/refractory solid tumor malignancies or lymphoma.
NCT02736565	DOTAP:Chol	Pbi‐shRNA™ EWS/FLI1 Type 1	Ewing's sarcoma	I (2016)	Gradalis, Inc. (New York, NY, USA)	This clinical trial aims to determine the maximum tolerated dose and safety of pbi‐shRNATM EWS/FLI1 Type 1 lipoplex in patients with advanced Ewing's sarcoma.
NCT01808638	AtuFECT01‐DPhyPE/DSPE‐PEG‐2000	PKN3 siRNA	Pancreatic cancer	I/II (2013)	Silence Therapeutics	The study aims to assess a novel treatment approach for advanced pancreatic cancer. It will use a new investigational drug called Atu027 in combination with the standard chemotherapeutic drug gemcitabine to enhance gemcitabine's antitumor activity.
NCT01505153	DOTAP/Chol	bi‐shRNA‐stathmin 1 pDNA	Solid tumors	I (2004)	University of Pittsburgh	This is a safety trial of pbi‐shRNA™STMN1 lipoplex administered by intratumoral injection.
NCT03338777	DMRIE/DOPE	(HSTK, cIFNβ, hIL‐2, hGM‐CSF) pDNA	Melanoma	I (2017)	Hospital Italiano de Buenos Aires	This clinical trial protocol aims to assess the safety of combined genetic and immunotherapy in humans.
NCT01591356	DOPC	EphA2 siRNA	Advanced malignant solid neoplasm	I (2012)	M.D. Anderson Cancer Center (Houston, TX, USA)	This clinical trial aims to assess the safety and determine the optimal dosage of EphA2 siRNA in the treatment of patients with advanced or recurrent solid tumors that have metastasized to other parts of the body and are usually untreatable or cannot be controlled with available therapies.

*Data source*, ClinicalTrials.gov website.

## 7 CONCLUSIONS AND REMARKS

Nanoliposome‐based cancer gene therapy represents a promising approach to address the limitations of traditional cancer treatments. The multifaceted nature of nanoliposomes allows for precise control over drug delivery, enabling targeted and efficient delivery of therapeutic agents to tumor sites while minimizing systemic toxicity. However, despite the considerable progress made in this field, several critical challenges remain to be addressed to facilitate the clinical translation of nanoliposome‐based therapies. One such challenge is the optimization of nanoliposome design to improve stability, specificity, and payload capacity. Advances in lipid composition, surface modifications, and targeting ligands hold the potential to enhance the therapeutic efficacy of nanoliposomes and overcome existing limitations.

Moreover, the safety profile of nanoliposome‐based therapies must be thoroughly evaluated to ensure their clinical applicability. Immunogenicity, long‐term toxicity, and potential adverse effects on healthy tissues are essential considerations that need to be addressed through comprehensive preclinical studies and clinical trials. Furthermore, the development of innovative strategies to mitigate immunogenicity and enhance the biocompatibility of nanoliposomes will be essential for their widespread adoption in clinical practice.

In addition to addressing safety concerns, future research efforts should focus on harnessing the synergistic effects of codelivering multiple therapeutic agents using nanoliposomes. Combination therapies, incorporating gene constructs, chemotherapeutic drugs, and immunomodulatory agents, have the potential to significantly enhance therapeutic outcomes and overcome treatment resistance in cancer patients. Furthermore, the integration of advanced gene editing technologies, such as CRISPR/Cas9, offers exciting opportunities for precise targeting and modulation of cancer‐related genes, paving the way for personalized and tailored treatments.

Overall, nanoliposome‐based cancer gene therapy holds immense promise for revolutionizing cancer treatment paradigms. By addressing existing challenges and embracing emerging technologies, nanoliposomes have the potential to transform the landscape of cancer therapy and improve patient outcomes. Continued collaboration between researchers, clinicians, and industry stakeholders is essential to drive innovation, advance clinical development, and ultimately bring nanoliposome‐based therapies to the forefront of cancer treatment. Through concerted efforts, nanoliposomes have the potential to fulfill their promise as a powerful tool in the fight against cancer, offering hope to patients and their families worldwide.

## AUTHOR CONTRIBUTIONS

*Writing—original draft*: Safiye Nur Yildiz, Maliheh Entezari, Mahshid Deldar Abad Paskeh, Sepideh Mirzaei, Alireza Kalbasi, Amirhossein Zabolian, Farid Hashemi, Kiavash Hushmandi, Mehrdad Hashemi, Mehdi Raei, and Mohammad Ali Sheikh Beig Goharrizi. *Visualization*: Safiye Nur Yildiz and Yavuz Nuri Ertas. *Writing—reviewing*: Amir Reza Aref, Ali Zarrabi, Jun Ren, Gorka Orive, Navid Rabiee, and Yavuz Nuri Ertas. *Editing and supervision*: Yavuz Nuri Ertas. All the authors have read and approved the final version of the manuscript.

## CONFLICT OF INTEREST STATEMENT

Author Amir Reza Aref is an employee of Xsphera Biosciences, Inc. Author Gorka Orive is an employee of Bioaraba, NanoBioCel Research Group. Other authors have no interest to declare.

## ETHICS STATEMENT

Not applicable.

## Data Availability

Not applicable.
